# Stimulation of C-Kit^+^ Retinal Progenitor Cells by Stem Cell Factor Confers Protection Against Retinal Degeneration

**DOI:** 10.3389/fphar.2022.796380

**Published:** 2022-03-31

**Authors:** Xi Chen, Shanshan Li, Xiaoli Liu, Jingjie Zhao, Lanting Wu, Ran You, Yanling Wang

**Affiliations:** ^1^ Department of Ophthalmology, Beijing Friendship Hospital, Capital Medical University, Beijing, China; ^2^ Department of Pediatric Newborn Medicine, Brigham and Women’s Hospital and Harvard Medical School, Boston, MA, United States; ^3^ Department of Traditional Chinese Medicine, Beijing Friendship Hospital, Capital Medical University, Beijing, China

**Keywords:** retinal degeneration, c-kit, stem cell factor, retinal ganglion cell, crystallins

## Abstract

C-kit/CD117, expressed in a series of tissue-specific progenitor cells, plays an important role in tissue regeneration and tissue homeostasis. We previously demonstrated that organoid-derived c-kit^+^ retinal progenitor cells can facilitate the restoration of degenerated retina. Meanwhile, we have identified a population of endogenous c-kit^+^ cells in retinas of adult mouse. However, the exact role of these cells in retinal degeneration remains unclear. Here, we demonstrated that stimulation of endogenous c-kit^+^ cells by stem cell factor (SCF) conferred protection against retinal degeneration. Retinal degeneration was induced by intravitreal injection of N-methyl-D-aspartate (NMDA). NMDA challenge increased the total number of c-kit^+^ cells in the retinal ganglion cell layer (GCL), while deregulated the protein level of SCF, which was mainly expressed in Müller cells. Both flash electroretinogram (fERG) and light/dark transition tests showed that intravitreal injection of SCF effectively improved the visual function of NMDA-treated mice. Mechanistically, SCF administration not only prevented the loss of retinal ganglion cells (RGCs), but also maintained the function of RGCs as quantified by fERG. Further, we performed transcriptome sequencing analysis of the retinal cells isolated from SCF-treated mice and the parallel control. Gene Ontology analysis showed that SCF-induced transcriptome changes were closely correlated with eye development-related pathways. Crystallins and several protective factors such as *Pitx3* were significantly upregulated by SCF treatment. Our results revealed the role of SCF stimulated c-kit^+^ cells in the protection of RGCs in NMDA-treated mice, *via* inhibiting the loss of RGCs. Administration of SCF can act as a potent strategy for treating retinal degeneration-related diseases.

## Introduction

The death of neurons is the leading cause of blindness in retinal degeneration (Pascolini and Mariotti, 2012; Wong et al., 2014). For example, in patients with glaucoma, retinal ganglion cells (RGCs), the neurons in the mammalian retina, undergo progressive degeneration, which leads to an irreversible vision loss (Cuenca et al., 2014). Rescue of retinal neurons has been considered as an effective strategy for the retina regeneration therapy (Jin et al., 2019; Singh et al., 2020). Stem/progenitor cell transplantation can be differentiated into retinal neurons, while the preparation of transplantable cells is inevitably complicated (Schwartz et al., 2015; Schwartz et al., 2016; Mandai et al., 2017). Reprogramming Müller cells can also regenerate retinal neurons *via* virus-mediated genome editing, while it can yet be applied due to safety issues (Jorstad et al., 2017; Yao et al., 2018; Hoang et al., 2020; Jorstad et al., 2020; Zhou et al., 2020). Neuroprotective factors, such as brain-derived neurotrophic factor (BDNF), can promote cell survival and prevent retinal neuron death, with some drawbacks such as short half-lives of these factors and the inability to cross the blood-retina barrier. Therefore, to develop a simple, safe and effective strategy to facilitate the repair of injured retina remains a key challenge.

In the previous study, we have demonstrated that c-kit^+^ retinal progenitor cells (RPCs) would be a promising cell source for repairing the injured retina. By enriching c-kit^+^ RPCs from human embryonic stem cell-derived retinal organoids, we found that subretinal transplantation of c-kit^+^ RPCs into retinal degeneration models could significantly improve vision and delaying retinal degeneration (Zou et al., 2019). Moreover, we have previously identified a population of c-kit^+^ cells in retinas of both postnatal and adult mouse, especially containing regenerative potential during adulthood (Zhou et al., 2015; Chen et al., 2016; Chen et al., 2017). However, the exact role of these c-kit^+^ cells in the retina remains unclear.

Here, we reported that activation of endogenous c-kit^+^ cells can protect against retinal degeneration. In pathological conditions, such as N-methyl-D-aspartate (NMDA) challenge, the total number of c-kit^+^ cells in the retina was increased, while the expression level of SCF was downregulated. Supplementation of exogenous SCF can effectively facilitate the preservation of the retinal function, by inhibiting the loss of RGCs. Transcriptome analysis showed that several eye development-related factors, such as Crystallins, were significantly upregulated by SCF treatment. In summary, our study demonstrated the protective role of SCF/c-kit signaling on the retinal injury, and indicated exogenous SCF as a potent candidate for the treatment of retinal degeneration-related diseases.

## Materials and Methods

### Mice

*C57BL/6J* mice were provided by the Institutional Animal Care of Beijing Friendship Hospital Affiliated to Capital Medical University. Four-week-old mice (males and females) were randomly assigned to groups, and maintained under a standard 12-h light/dark cycle at 24.5°C. All experimental procedures were approved by the Office of Research Ethics Committee at Beijing Friendship Hospital Affiliated to Capital Medical University (ethics approval number: 18-2020).

### Intravitreal Injections

Animals were anaesthetized with 1.5–2% isoflurane. Intravitreal injection of NMDA (100 mM in PBS, 2 μl per eye; Cat# M3262, Sigma-Aldrich, United States) was performed using a sharp 32-guage needle (micro-syringe equipped of Hamilton Storage, United States). Two days post NMDA challenge, intravitreal injection of recombinant SCF (50 ng/μl in PBS, 2 μl per eye; Cat# 455-MC, Novus Biologicals, United States) and SU 5416 (c-kit inhibitor, ic-kit, 50 ng/μl in PBS, 2 μl per eye; Cat# 3037, Tocris Bioscience, United Kingdom) were performed. Mice injected with an equal volume of PBS (2 μl per eye) were served as control.

### Tissue Preparation and Immunofluorescence

Immunohistochemistry was performed as described previously (Chen et al., 2016; Chen et al., 2017). Briefly, mouse eyeballs were prefixed in prefixation buffer (5% acetic acid, 0.4% paraformaldehyde, 0.315% saline, and 37.5% ethanol), then incubated in 4% paraformaldehyde overnight at 4°C, followed by embedded in paraffin. Eyecups were sectioned at 5 μm on a microtome (Leica, Germany). Slides were then deparaffinized, rehydrated, and boiled in 10 mM citrate buffer, followed by incubation in 5% donkey serum for 30 min at room temperature. Slides were then incubated with indicated primary antibodies at 4°C overnight, rinsed with PBS, and then incubated in species-matched fluorophore-conjugated secondary antibodies for 1 h at 37°C. Nuclei were counterstained with 4’,6-diamidino-2-phenylindole (DAPI). Images were obtained using confocal microscopy of Fluo View FV1000 (Olympus, Japan). To perform the immuno-positive cell quantification, immuno-positive cells were counted in sections of the whole retina from the nasal side to the temporal side across the optic disk, and at lease six sections per mouse were analyzed. The numbers of immuno-positive cells obtained of each section from one mouse were averaged, following the protocol described previously (De la Huerta et al., 2012; Li et al., 2016; Zou et al., 2019).

The primary antibodies used were as follows: anti-c-kit at 10 μg/ml (Cat# AF1356, R&D Systems, United States), anti-glutamine synthetase (GS) at 1:200 (Cat# ab73593, ab64613; Abcam, United Kingdom), anti-SCF at 1:200 (Cat# ab64677; Abcam), anti-Connexin 43 (Cx43) at 1:100 (Cat# ab78055; Abcam), anti-Iba1 at 1:200 (Cat# ab178847; Abcam), anti-NeuN at 1:200 (Cat# ab209898; Abcam), and anti-Calretinin at 1:400 (Cat# MAB1568; Millipore). The secondary antibodies used were as follows: donkey anti-goat IgG Alexa Fluor 488 at 1:500 (Cat# ab150129; Abcam), donkey anti-rabbit Alexa Fluor 555 at 1:500 (Cat# ab150074; Abcam), donkey anti-mouse Alexa Fluor 555 at 1:500 (Cat# ab150106; Abcam), donkey anti-mouse Alexa Fluor 647 at 1:500 (Cat# ab150107; Abcam), donkey anti-rabbit IgG Alexa Fluor 488 at 1:500 (Cat# ab150073; Abcam), goat anti-chicken IgG Alexa Fluor 555 at 1:500 (Cat# ab150170; Abcam).

### Analysis of Microglia

The method was performed as described previously (Zou et al., 2019). Briefly, five 40× field views were captured from three 15 μm-thick retinal sections per eye using the Olympus confocal imaging system with 1-μm z-steps. By using a grid system, the number of grid-crossing points per individual microglia cell was counted (*n* > 3 eyes per group). The number of Iba1^+^ cells was counted in five eyes per group.

### Electroretinogram Recording

Corneal scotopic flash electroretinogram (fERG) of mice was performed at corresponding time point after intravitreal injection of NMDA (at lease five mice in each time point) as described previously (Chen et al., 2016). Briefly, after adaption darkness overnight, mice were anesthetized with 1.5–2% isoflurane. The animal body temperature was maintained at 37°C by using a heating pad. The pupils of mice were dilated with tropicamide and phenylephrine eye drops (Santen Pharmaceutical, Japan). The recording electrodes of gold loops were placed on the cornea. The reference electrodes and grounding electrodes of gold needles were inserted subcutaneously into angulus oculi and tail respectively. We obtained flash recordings at the light intensities of −2.5, −0.5, −0.02, 0.5 and 2.5 log (cd*s/m^2^) using Reti-scan system (Roland Consult, Germany). Waves measured at 2.5 log10 (cd*s/m^2^) were presented. The fERG procedures were performed under the environment of dim red light. The amplitudes of a-wave and b-wave were analyzed among groups.

Scotopic threshold responses (STRs) were elicited using a −4.5 log10 (cd*s/m^2^) stimulus using Reti-scan system (Roland Consult) as described previously (Holcombe et al., 2008). Thirty flashes with an interstimulus interval of 2s were averaged. Amplitudes of the positive STR (pSTR) and negative STR (nSTR) were measured for about 140 and 220 ms after the stimulus flash, respectively.

For photopic negative response (PhNR) analysis, flash strength was 10 log10 (cd*s/m^2^), and 50 responses were averaged for each eye as described previously (Jnawali et al., 2020). The PhNR was measured from baseline to the trough immediately following the b-wave.

### Light/Dark Transition Test

Light/dark transition test was performed as described previously (Chen et al., 2016). The light/dark box consists of one light chamber (45 × 30 × 40 cm) and one dark chamber (15 × 30 × 40 cm), and these two compartments were connected with a door (10 × 10 cm). Mice were maintained in dark environment overnight, and adapted in the dark chamber for 2 min. The door was then opened, and mice were allowed to freely move into the light chamber for 5 min with 300 lux of tungsten filament bulb over the center of the compartment. All of the mice were tested naïve (only one test per mouse). Four paws completely through the door were defined as entering the light chamber. The time of exploratory behavior in the light compartment was analyzed.

### Western Blotting

Eye samples were prepared after mice with euthanized. Retinas were then isolated and homogenized in an ice-cold mixture of RIPA buffer (Beyotime, China) containing protease inhibitor cocktail (Beyotime). Extracts were separated using 12% sodium dodecyl sulfate poly-acrylamide gels and transferred onto polyvinylidene fluoride membranes. Membranes were incubated in TBST (12.5 mM Tris–HCl, pH 7.6, 75 mM NaCl, 0.1% Tween 20) containing 5% fat-free milk for 1 h at room temperature, then transferred into solution containing primary antibodies at 4°C overnight, and probed with indicated secondary antibodies in TBST for 2 h at room temperature. Membranes were exposed on an Odyssey infrared imaging system with the Odyssey Application software V1.2.15 (LI-COR Biosciences, United States). All blots were analyzed by ImageJ (National Institutes of Health, United States). The relative levels of SCF were determined by normalizing against β-actin. The primary antibodies used were as follows: anti-SCF at 1:1000 (Cat# ab64677; Abcam), anti-β-actin at 1:1000 (Cat# ab179467; Abcam). The secondary antibody used was peroxidase-conjugated goat anti-rabbit IgG at 1:2000 (Beyotime).

### Flow Cytometry

Flow cytometry was used to identify c-kit^+^ cells and was performed as described previously (Chen et al., 2016; Chen et al., 2017). Briefly, the retinas were dissociated in PBS containing collagenase I (10 mg/ml) and collagenase II (25 mg/ml, Worthington Biochemical, United States). The dissociated cells were filtered through a 40-μm filter (BD Biosciences, United States) and then blocked with CD32/16 for 15 min at room temperature. Then, the cell suspensions were incubated with c-kit antibody conjugated with APC fluorescence (1:50; Cat# 17-1172-83; eBioscience, Thermo Fisher Scientific, United States) or isotype control (1:50; Cat# 17-4031-82; eBioscience) for 30 min at 4°C. After each procedure, cells were rinsed with staining buffer (eBioscience). Cells were counted using a FACSCalibur Flow Cytometer and at least 10,000 events were collected for each sample and analyzed using FlowJo software (FlowJo, United States).

### Cell Culture

Thec-kit^+^ cells were sorted from retinal cells of postnatal day 1 pups after cultured for one passage by fluorescence-activated cell sorting, then were cultured in medium containing DMEM/F12 medium (Lonza Biologics, United States) supplemented with 10% fetal bovine serum (Thermo Fisher Scientific, United States), 20 ng/ml murine basic fibroblast growth factor (PeproTech, United States), 20 ng/ml murine epidermal growth factor (PeproTech), insulin/transferrin/sodium selenite (1:500; Lonza Biologics), and 10 ng/ml leukemia inhibitor factor (EMD Millipore, United States) as described (Chen et al., 2016). BV2 mouse microglia cells were cultured in high-glucose DMEM supplemented with 10% FBS. For *in vitro* co-culture assay, BV2 microglia cells and c-kit^+^ cells were cultured separately at a density of 1 × 10^5^ cells/well for 6 h, and then treated with 1 μg/ml LPS or 50 ng/ml SCF respectively for 24 h. The LPS-treated BV2 cells were seeded on 24-well cell culture inserts, and co-cultured with SCF-treated C-Kit^+^ cells seeded in the lower chamber (EMD Millipore) at a density of 10^3^–10^5^ per well for 24 and 48 h. Monocultures of microglia with or without LPS treatment were served as controls. All of the cultures were maintained under consistent and equivalent conditions. The experiments were performed in triplicate, and each result represents the mean of three independent experiments.

### Gene Functional Annotation Analysis

For transcriptome analysis, total retinal cells were incubated in RNAiso Plus (Takara, Japan) at a concentration of 2 × 10^6^ cells/ml and stored at −80°C. All samples were transported to the Genomics Institute on dry ice for the transcriptome study. The mRNA sample was enriched using oligo (dT) magnetic beads and fragmented into short fragments using fragmentation buffer. The corresponding cDNA libraries were produced and qualified using an Agilent 2100 Bioanalyzer and an ABI StepOnePlus Real-Time PCR System. Primary raw reads produced by HiSeq 4000 (Illumina, United States) were qualified and filtered to obtain clean reads. A heatmap analysis of gene expression levels were created based on the averaged fragments per kilobase of exon per million fragments mapped (FPKM) values of genes. Genes with fold change ≥2 and adjusted *p* values ≤0.001 were considered as the differentially expressed genes (DEGs). Annotation analysis of Gene Ontology (GO) was performed to determine the on-going biological process. RNA-seq data were deposited in the Gene Expression Omnibus (GSE192458).

### RNA Isolation and Real-time Quantitative Polymerase Chain Reaction (PCR)

Total RNA was purified using TRIzol reagent (Invitrogen, United States) followed by chloroform extraction. After reverse transcription performed using the PrimeScriptTM RT reagent kit with gDNA Eraser (Takara, Japan), real-time qPCR reactions were performed using a CFX96 Real-time qPCR System (Bio-Rad, United States) using SYBR^®^ Premix Ex TaqTM II (Takara) to measure the expression of various genes. The level of *GAPDH* mRNA expression was used to normalize differences in the levels among different transcripts. The primers used are listed in Supplementary Table S1.

### Statistical Analysis

All statistical differences were performed on SPSS 23.0 by one-way ANOVA test among comparisons groups. Data are presented as mean ± standard deviation (SD). Differences were considered as significant at *p* < 0.05.

## Results

### Increased Number of C-Kit^+^ Cells in NMDA-Treated Mice

**FIGURE 1:**
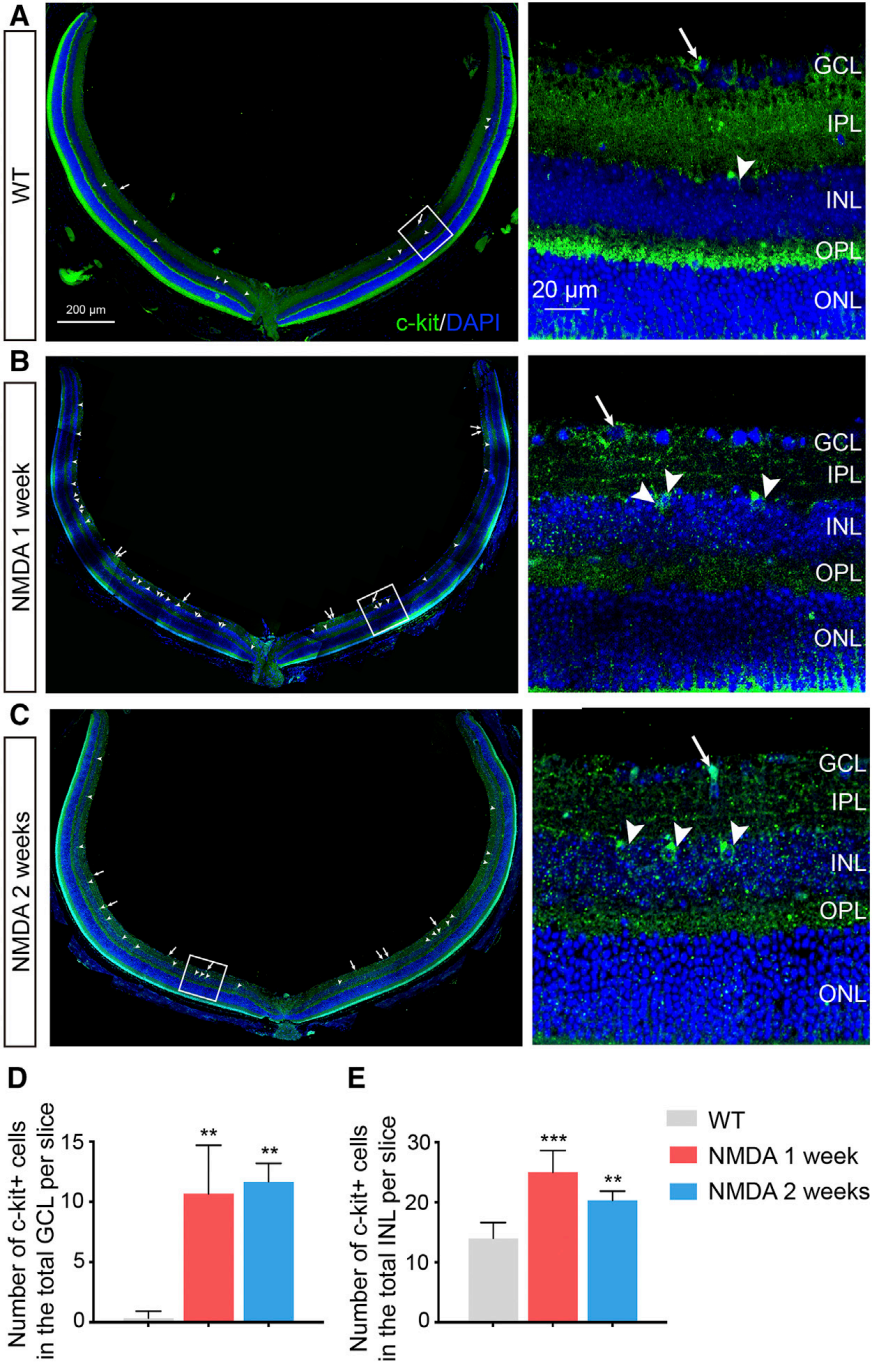
Increased Number of C-kit^+^ Cells in NMDA-treated Mice. **(A–C)** Representative images of c-kit^+^ cell staining (green) in retinas of indicated mice with NMDA exposure. Area in the white boxes **(A** **–** **C)** was shown at higher magnification and displayed to the right. White arrow, the c-kit^+^ cell body in the retinal ganglion cell layer (GCL); white arrowhead, the c-kit^+^ cell body in the inner nuclear layer (INL). DAPI, 4′, 6-Diamino-2-Phenylendole; ONL, outer nuclear layer. Scale bars represented 200 μm of left panel, 20 μm of right panel. **(D,E)** The total number of c-kit^+^ cells in the GCL **(D)** and INL **(E)** were then quantitated and plotted as the mean ± SD per slice. *n* ≥ 3 for each time point, *p < 0.05 vs WT, **p < 0.01, ***p < 0.001.

C-kit, also known as CD117, is a type III receptor tyrosine kinase expressed in various types of stem cells, such as hematopoietic stem cells (Edling and Hallberg, 2007; Lennartsson and Ronnstrand, 2012). C-kit^+^ RPCs transplantation has been demonstrated as a potential strategy to improve vision and delay retinal degeneration. We have previously identified a population of c-kit^+^ RPCs in the retinas of both postnatal and adult mice (Chen et al., 2016; Chen et al., 2017). Further, in c-kit-Cre LacZ mice, the expression of β-galactosidase was restricted to RGCs and amacrine cells in the retina, suggesting these c-kit^+^ cells may differentiate into retinal neurons (Harada et al., 2011). However, the biological significance of these endogenous c-kit^+^ RPCs in the degenerative retina remains unclear. To elucidate this issue, we examined the distribution and abundance of c-kit^+^ cells in retina of the retinal degeneration mice model generated by NMDA injection. Consistent with previous reports**,** administration of NMDA led to a loss of RGCs and a decrease in the thickness of the inner plexiform layer (IPL), both of which are typical symptoms of retinal degeneration (Figures 1A–C).

We next examined the distribution and abundance of c-kit^+^ cells in retina of these retinal degeneration mice by immunohistochemistry. The morphology and distribution of c-kit^+^ cells were not markedly affected by NMDA treatment (Figures 1A–C). However, an increase in the number of c-kit^+^ cells in both retinal ganglion cell layer (GCL) and inner nuclear layer (INL) were observed in retinas after 1 week post NMDA challenge, compared with that in the wild-type (WT) retinas, and the effect was sustained for at least 2 weeks (*p* < 0.05; Figures 1D,E). These data confirmed that c-kit^+^ cells indeed existed in both GCL and INL in the retina.

The tissue was dissociated into single cell suspensions, and the presence of c-kit^+^ cells was established by flow cytometry (Supplementary Figure S1). Consistent with the results by immunofluorescence staining, the percentages of c-kit^+^ cells in the entire retina were notably higher after NMDA treatment (*p* < 0.001), especially 1 week post NMDA challenge. The increased number of c-kit^+^ cells in the retina of NMDA-treated mice suggested that these cells might be involved in retinal degeneration process.

### The Expression of SCF Was Downregulated After NMDA Treatment

**FIGURE 2:**
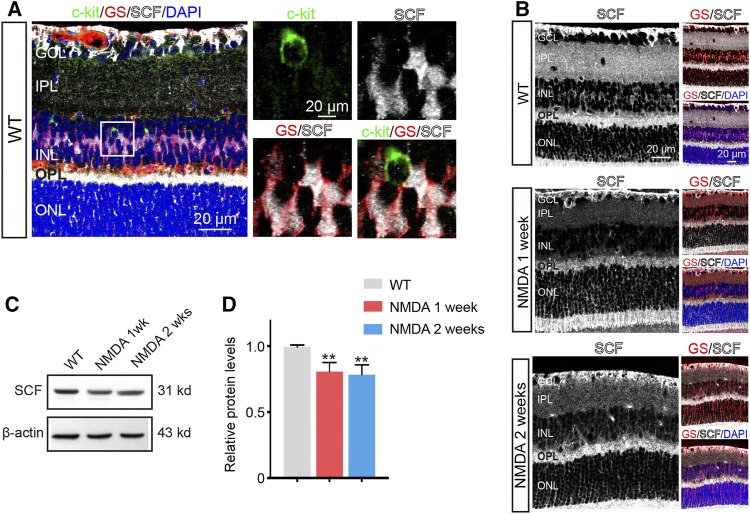
The Expression of SCF was Downregulated after NMDA Treatment. **(A)** c-kit-positive cells (green), glutamine synthetase (GS)-positive cells (red) and SCF-positive cells (white) located in the inner nuclear layer (INL). The white box in left panel was shown at higher magnification in right panels. **(B)** Immunofluorescence staining of SCF (white) and GS (red) in the retinas of WT and NMDA-treated mice. Scale bars represented 20 μm. **(C,D)** The expression of SCF were analyzed by Western blotting analysis **(C)** and quantitated by ImageJ and plotted as the mean expression ± SD (*n* = 3 eyes/group, **p < 0.01 vs WT) **(D)**. The level of SCF was corrected for β-actin loading control and normalized to the levels of control cells.

C-kit can be activated by its ligand stem cell factor (SCF), a growth factor that exists as a soluble or membrane-bound form (Cardoso et al., 2017). The functional SCF/c-kit signaling is critical for the survival and development of stem cells in hematopoiesis, pigmentation and reproduction (Lennartsson et al., 2005). We thus examined the expression of SCF in mouse retinas. In the retina of WT mice, SCF-expressing cells were mainly localized in INL as well as inner limiting membrane, adjacent to c-kit^+^ cells (Figure 2A). These SCF-positive cells can also express glutamine synthetase (GS), a marker of Müller cells, suggesting that in the INL, Müller cells act as an endogenous source of SCF. Also, the gap junction protein Connexin 43 (Cx43) was distributed between c-kit^+^ cells and SCF-expressing Müller cells (Supplementary Figure S2).

As NMDA challenge influenced the abundance of c-kit^+^ cells, we then examined the expression of SCF in retinas with NDMA stimulation. Comparing to WT mice, the expression of SCF in retinas was downregulated after NMDA exposure, obtained by immunofluorescence and Western blot analysis (Figures 2B–D), and also confirmed by real-time qPCR (Supplementary Figure S3). Together with the previous observation showing the increased number of c-kit^+^ cells (Figures 1D,E), these data indicated that the insufficient activation of SCF/c-kit signaling may be involved in the progression of NMDA-induced retinal degeneration.

### Exogenous SCF Treatment Improved Visual Function of NMDA-Treated Mice

**FIGURE 3:**
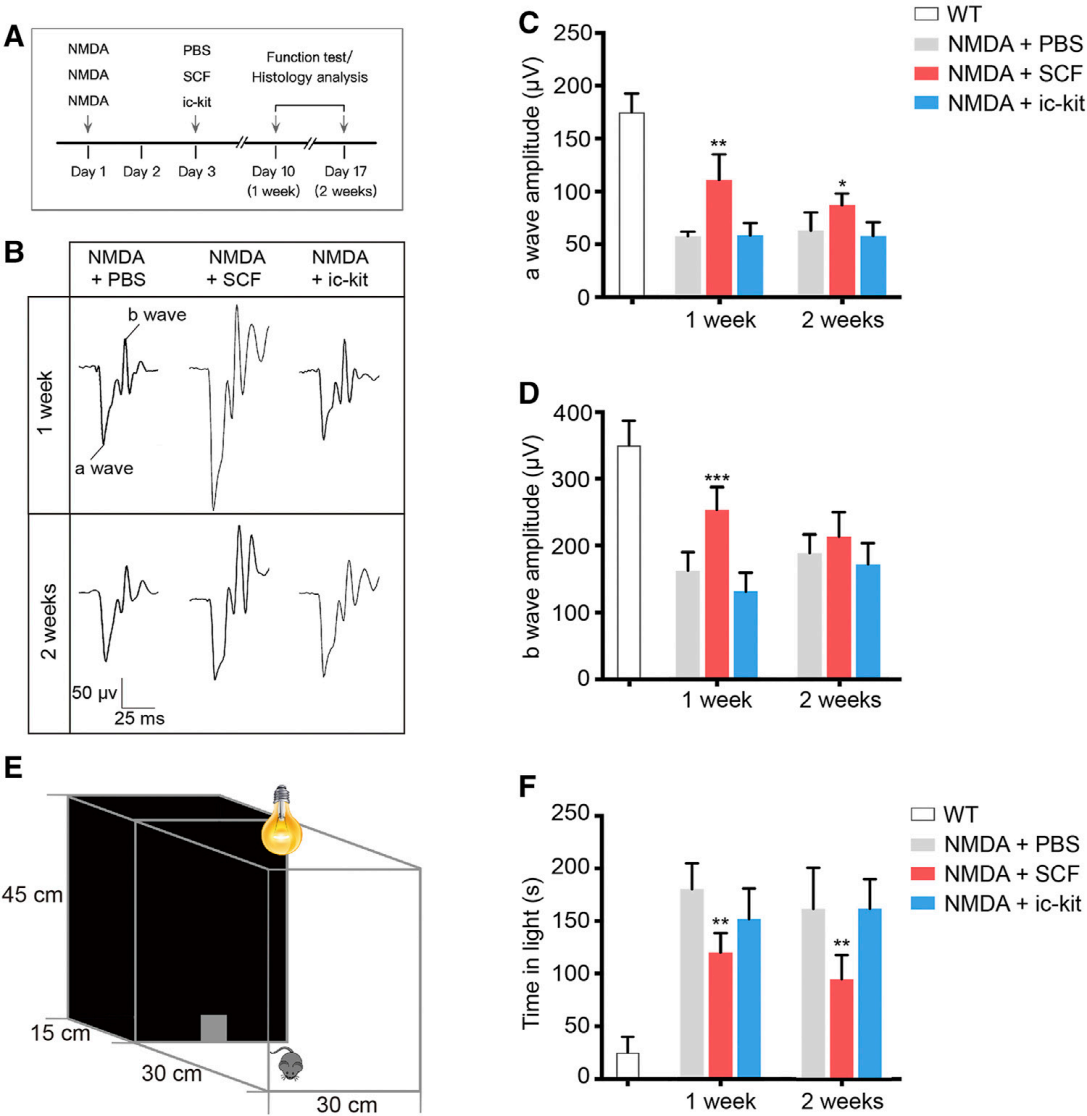
Exogenous SCF Treatment Improved Visual Function of NMDA-treated Mice. **(A)** Scheme of time points for intravitreal injections, function test and histology analysis. **(B)** Representative waves of three groups of mice measured through flash electroretinogram (fERG) tests at 2.5 log(cd*s/m^2^). **(C,D)** Statistical analysis of the amplitudes of fERG a-wave **(C)** and b-wave **(D)** in the indicated groups. **(E)** Diagram showing the setup of the light/dark transition test. **(F)** Time spent in the light compartment was recorded. Data were shown as the mean ± SD (*n* ≥ 5 for each time point). *p < 0.05, **p < 0.01, ***p < 0.001, compared with NMDA + PBS controls.

In view of the decreased SCF expression and increased number of c-kit^+^ cells in the NMDA-induced degenerative retinas, we wondered whether exogenous SCF supplementation can compromise the retinal degeneration and improve the vision function. To do so, recombinant SCF (50 ng/μl) was intravitreally administrated at 2 days post NMDA injection, while control group received same amount of PBS or c-kit inhibitor (ic-kit, 50 ng/μl) at the same timepoint after NMDA damage, respectively (Figure 3A).

To detect the retinal function of indicated mice, we performed flash electroretinogram (fERG) and the light/dark transition tests at 1- and 2-week post SCF administration (Figure 3). Mice receiving SCF showed markedly increased amplitudes of both the a-wave (Figures 3B,C) and b-wave (Figures 3B,D) at 0.5 log10 (cd*s/m^2^; the data for the other light intensity not shown), compared with PBS and ic-kit groups. Moreover, in the light/dark transition test (Figure 3E), mice receiving SCF injection for 1 and 2 weeks showed a behavioral aversion to light, and spent less time in the light chamber (Figure 3F), indicating that exogenous SCF improved retinal function of NMDA-treated mice.

**FIGURE 4:**
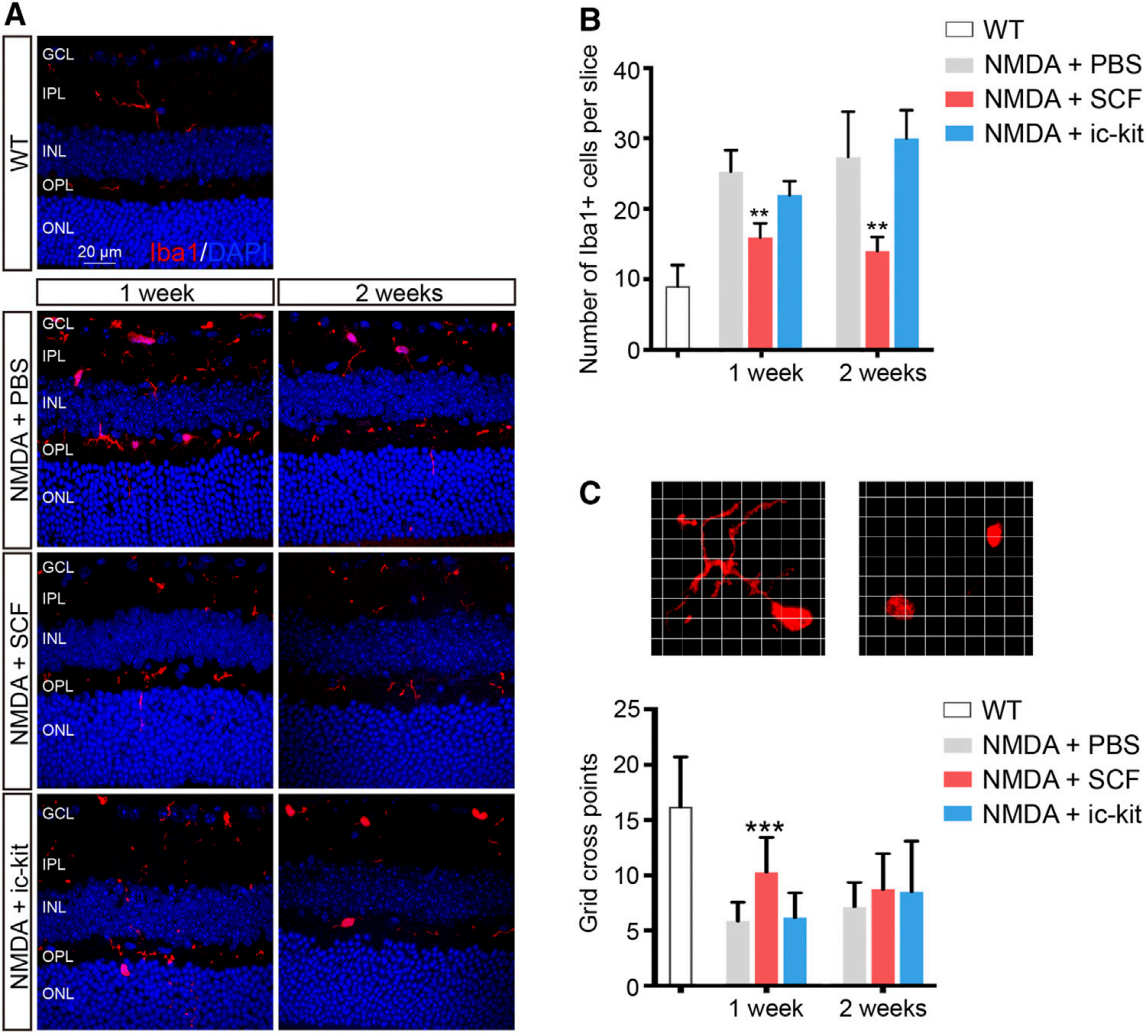
Activation of Microglia was Suppressed by SCF Supplementation. **(A)** Immunofluorescence detection of the morphology and distribution proportion of Iba1^+^ cells (red) in indicated mice. Scale bars represented 20 μm. **(B)** Number of Iba1^+^ cells were quantitated in each group (*n* ≥ 5 for each time point). **(C)** Representative images of ramified (left panel) or ameboid-like (right panel) Iba1^+^ microglia in grid crossing of each grid with a side length of 3 μm. Statistical analysis of the grid-crossing points per microglia (*n* ≥ 21 per group). The data were shown as mean ± SD. **p < 0.01, ***p < 0.001, compared with NMDA + PBS controls.

Microglia are considered as the major source of pro-inflammatory factors that contribute to retinal degeneration. Here we found that the activation of microglia has also been downregulated by SCF supplementation (Figure 4). Based on the results of Iba1 staining, reactive microglia were mainly distributed in the GCL, IPL and outer plexiform layer (OPL) in NMDA-treated retina (Figure 4A). However, in SCF-treated group, the number of activated microglia were significantly decreased, compared with control groups (*p* < 0.05; Figure 4B). The morphology change is another key feature for microglia activation, we thus quantified the microglia morphology using a grid cross-counting system as reported previously (Luckoff et al., 2016; Luckoff et al., 2017; Bian et al., 2020). By counting the grid-crossed points of Iba1^+^ cells, we found that SCF treatment markedly compromise the activation of the microglia. Moreover, the histogram data demonstrated that the microglia in the SCF-treated group mainly showed ramified shapes, while most microglia in the control group adopt an amoeboid morphology (Figure 4C). Also, it was demonstrated that SCF stimulated c-kit^+^ cells inhibited proliferation and pro-inflammatory factors expression of microglia *in vitro* (Supplementary Figure S4). Taken together, these data demonstrated that exogenous SCF treatment can inhibit the hyperactivation of microglia in the NMDA-treated retina, and thus improve the visual function.

### SCF Supplementation Compensated for the Loss of RGCs in NMDA-Treated Mice

**FIGURE 5:**
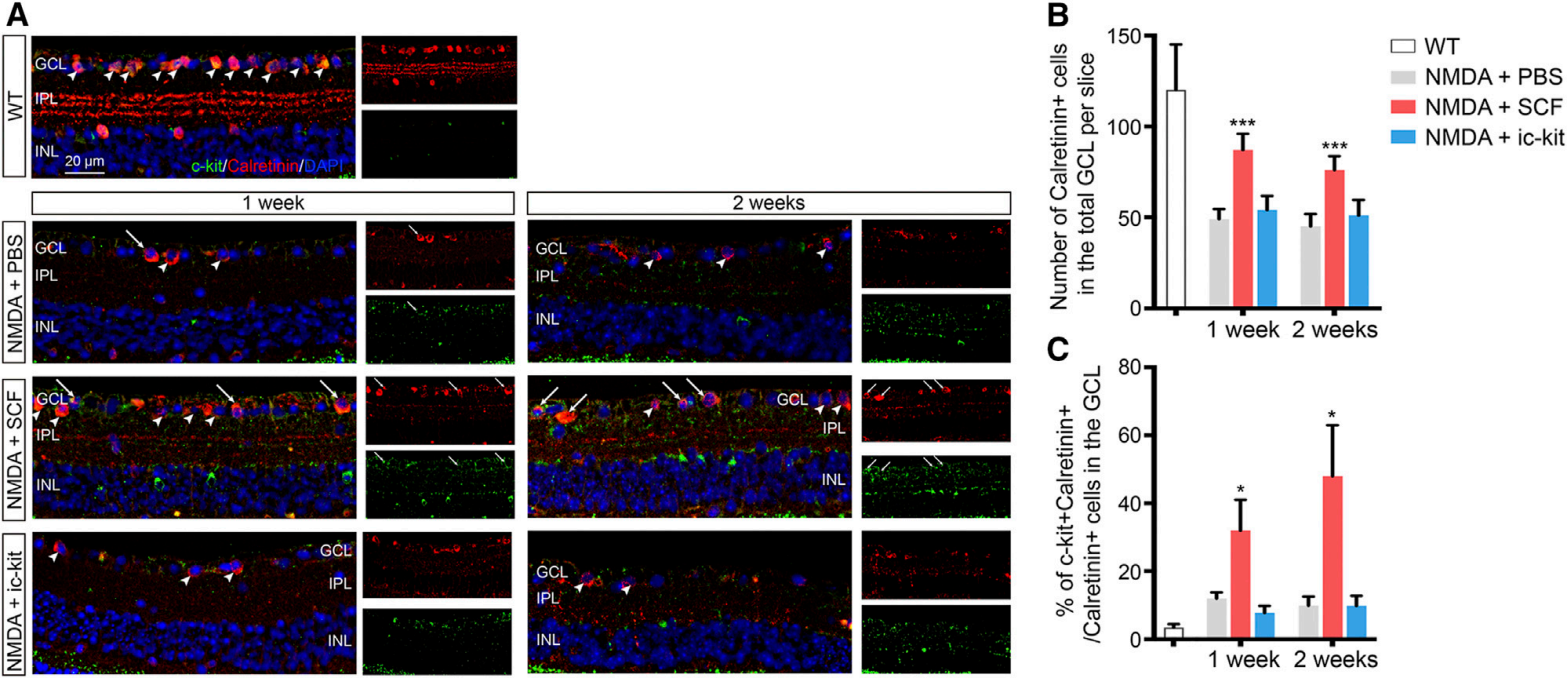
SCF Treatment Compensated for the Loss of RGCs in NMDA-treated Mice. **(A)** Immunofluorescence detection of Calretinin^+^ RGCs (red) in the GCL of indicated mice. Scale bars represented 20 μm. **(B)** Statistical analysis of the number of Calretinin^+^ RGCs in indicated groups. **(C)** Statistical analysis of the ratio of c-kit^+^ cells in Calretinin^+^ RGCs in indicated groups. Data were shown as mean ± SD (*n* ≥ 5 for each time point). *p < 0.05, **p < 0.01, ***p < 0.001, compared with NMDA + PBS controls.

The loss of RGCs, which can trigger the activation of microglia, is the leading cause of visual impairment in NMDA-induced retinal degeneration (Niwa et al., 2016). Since SCF supplementation can improve the NMDA-induce retinal degeneration, we then examined the protective role of SCF on RGCs. Both morphologic and functional assessments for RGCs were performed. As shown in Figures 5A,B and Supplementary Figure S5, the number of RGCs (indicated as Calretinin^+^ cells and NeuN^+^ cells) was markedly decreased after NMDA exposure. In line with previously observation (Figure 3), SCF treatment indeed attenuated the loss of RGCs. Further, we found that the administration of SCF increased the proportion of c-kit^+^ cells in GCL (Supplementary Figure S6). The majority of the increased RGCs also expressed c-kit (Figure 5C), indicating that after SCF treatment, c-kit^+^ cells may compensate for the loss of RGCs in NMDA mice.

**FIGURE 6:**
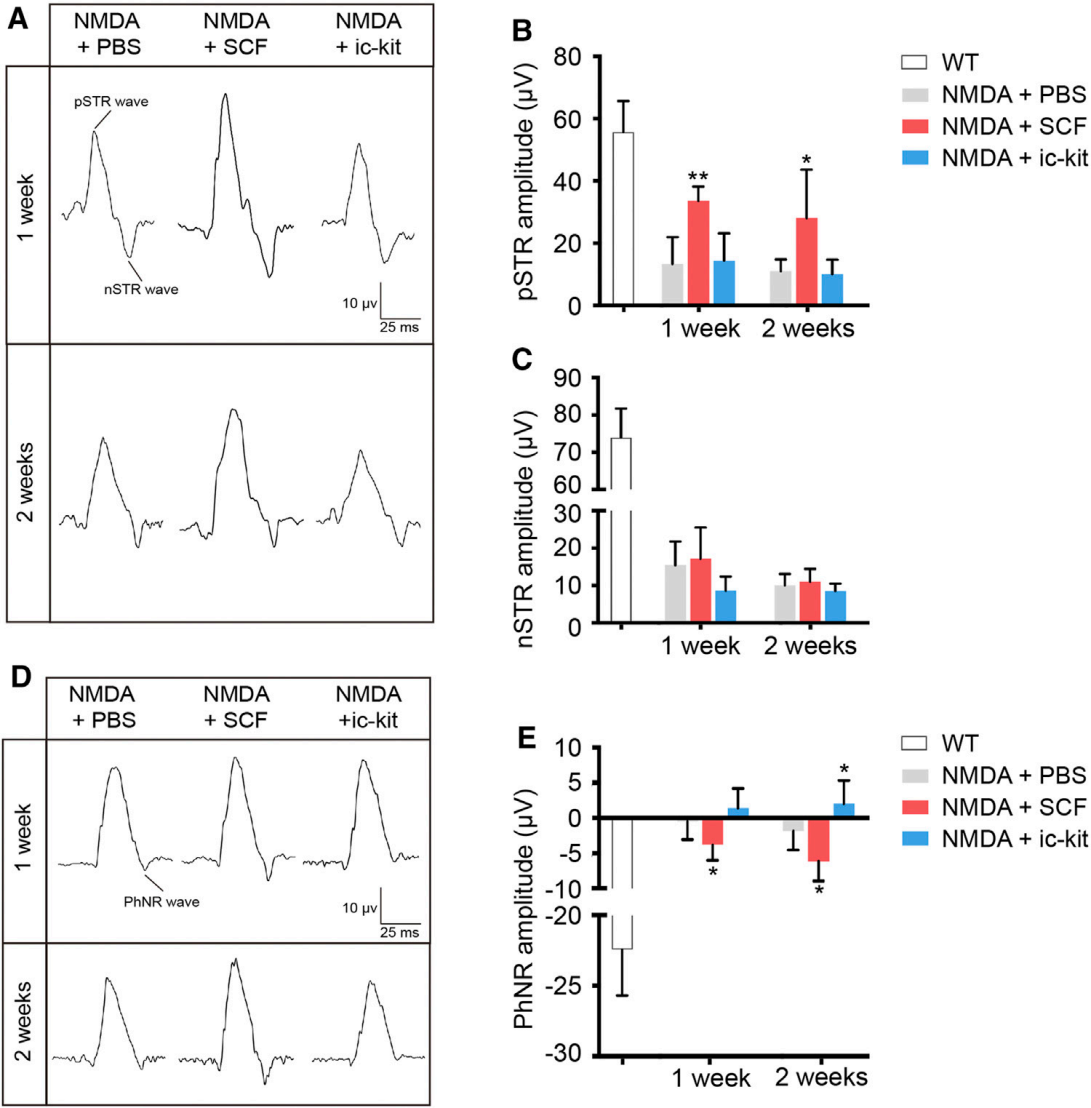
SCF Treatment Maintained the Function of RGCs in NMDA-treated Mice. **(A)** Specialized fERG tests for detecting the function of RGCs were performed at designed timepoints. Representative waves of positive scotopic threshold responses (pSTR) and negative STR (nSTR) in these groups. **(B,C)** Amplitudes of pSTR **(B)** and nSTR **(C)** were compared among groups. **(D)** Representative waves of photopic negative response (PhNR). **(E)** Statistical analysis of PhNR amplitudes among groups. Data were shown as mean ± SD (*n* = 5 for each time point). *p < 0.05, **p < 0.01, compared with the control group (NMDA + PBS).

We next examined the function of RGCs using specialized fERG. By comparing the ERG amplitudes recorded obtained from indicated groups, we found a significant increase in the pSTR of SCF-treated mice at 1- and 2-week post treatment (*p* < 0.05; Figures 6A–C). Compared to the control eyes, the PhNR amplitudes in the SCF-injected eyes were consistently increased, and stayed negative for at least 2 weeks (*p* < 0.05; Figures 6D,E). Taken together, these data demonstrated that exogenous SCF supplementation protected RGCs from NMDA-induced cell death, therefore delayed the progression of the retinal degeneration.

### RNA-Seq Reveals the Involvement of Key Genes for SCF Treatment

**FIGURE 7:**
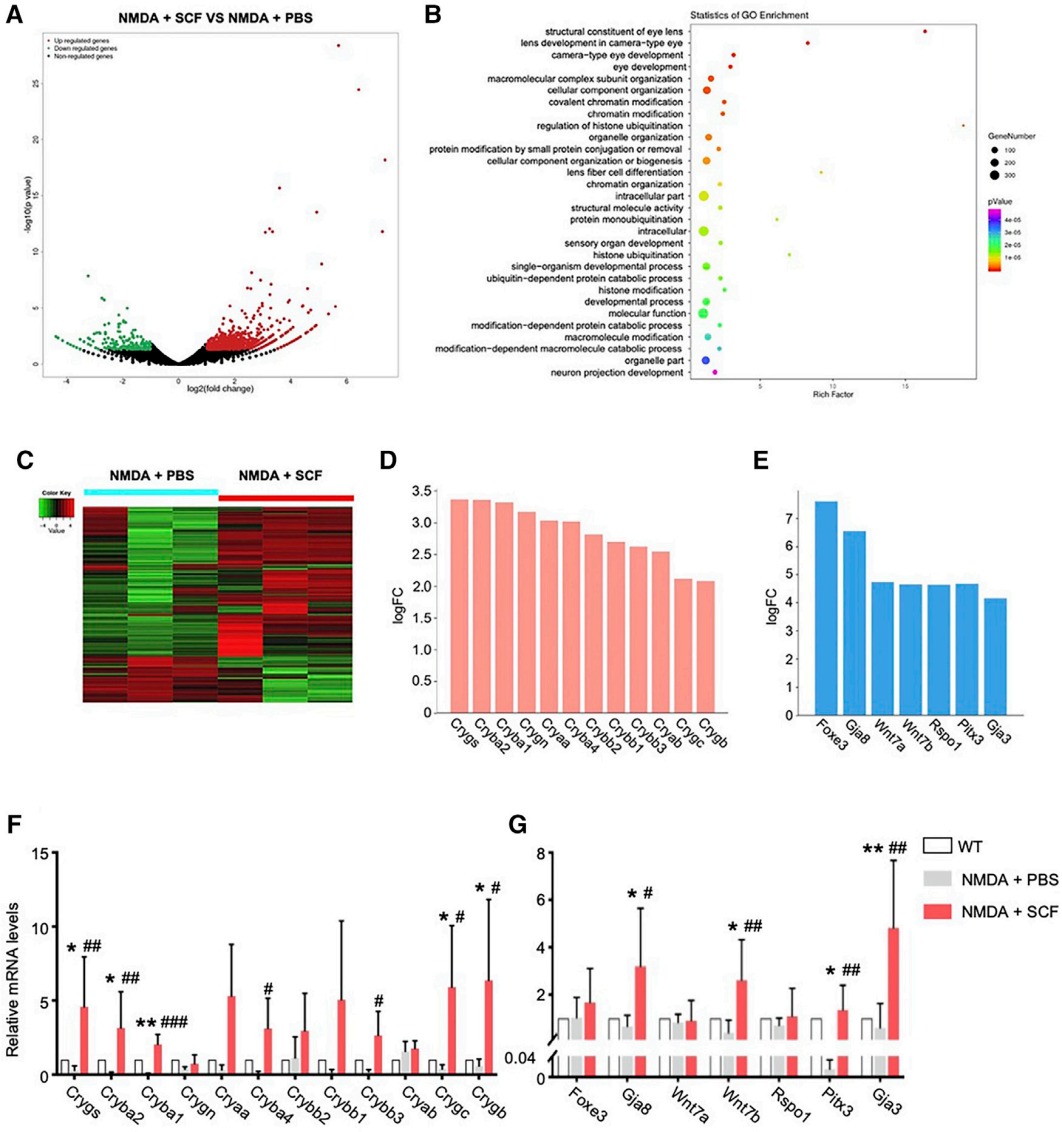
RNA-seq Revealed the Involvement of Key Genes for SCF Treatment. **(A)** Volcano plot of differentially expressed genes (DEGs) in SCF group compared with controls after 1 week treatment. **(B)** Gene Ontology (GO) analysis showing the enriched gene functions of SCF group versus control group. **(C)** Heatmap analysis showing DEGs in indicated groups. The relative abundance of each genus was indicated by a gradient of color from green (low abundance) to red (high abundance). **(D)** Crystallins family members were significantly up-regulated in SCF treated retinas. **(E)** Among top10 DEGs in SCF exposed mice, several neuro-protective factors were up-regulated, including Wnt7 related genes, *Foxe3/Pitx3*, and gap junction protein coding genes *Gja8*/*Gja3*. **(F)** Real-time qPCR analysis showing relative mRNA expression for the Crystallins family members among WT, NMDA +PBS and NMDA + SCF treated mice after 1 week. **(G)** Real-time qPCR analysis confirming the expression for the genes highly expressed in RNA-seq analysis. Data were shown as mean ± SD (*n* = 5 for each time point). *p < 0.05, **p < 0.01, compared with WT mice, #*p* < 0.05, ##*p* < 0.01, ###*p* < 0.001, compared with the NMDA + PBS group.

We next investigated the mechanism underlying the protective role of SCF/c-kit signaling on retinal degeneration. To do so, we employed a parallel transcriptome analysis by RNA sequencing. Retinal cells from SCF-treated mice after 1 week were isolated as described previously (Chen et al., 2017). A total of 359 genes were found to be differentially expressed in retinas from NMDA-treated mice with 1-week SCF treatment, compared with the cells derived from time-matched control retinas (NMDA plus PBS). The 359 differentially expressed genes (DEGs) included 287 upregulated genes and 72 downregulated genes (Figure 7A).

We then utilized the GO classification to analyzed the enriched pathways that were induced by SCF treatment. Accordingly, 359 DEGs identified in the present study were categorized into 14 functional groups (*p* < 0.00001). In the molecular function and biological process of GO classification categories, 1 and 13 functional groups were identified, respectively (Supplementary Table S2). SCF-treated retinas showed an enrichment in eye development-related pathways, including lens development, camera-type eye development, *etc.* (Figure 7B), suggesting SCF stimulated c-kit^+^ cells might contribute to RGC survival and retinal structure reconstruction.

To further analyze the DEGs, we found that the expression of multiple members from α, β and γ Crystallins family (*Cryaa*, *Cryab*, *Cryba1*, *Cryba2*, *Crygb*, *etc.*) were significantly up-regulated after SCF stimulation (Figures 7C,D). In addition, a series of factors that promote neuron survival were also significantly up-regulated with SCF exposure. The top10 up-regulated DEGs included *Pitx3*, *Foxe3*, *Gja3*, *Gja8*, *Wnt7a*, *Wnt7b*, *Rspo1*, *etc.* (Figure 7E). We confirmed these transcriptomic analysis results using qRT-PCR (Figures 7F,G). Taken together, these RNA-seq data revealed a change in the molecular signature of the retina, and suggested that the protective effect of SCF/c-kit pathway on NDMA-induced retinal degeneration may be mediated by both Crystallins and a series of protective factors.

## Discussion

Degenerative retinal disease is one of the leading causes of vision loss, while there are currently a limited number of effective treatments available (Ahmad et al., 2020). Here we found that stimulation of the endogenous c-kit^+^ cells by SCF compromised the NMDA-induced experimental retinal injury. Mechanistically, this effect, associated with the protection of RGCs, was possibly mediated *via* the upregulation of Crystallins and neuron-protective genes such as Pitx3, Foxe3 and Gja3. These finding suggested that applying SCF to stimulate endogenous c-kit^+^ cells would be an effective strategy for retina therapy.

Despite the controversy regarding the role of c-kit^+^ cardiac stem cells in the heart, various tissue-specific progenitor cells do express c-kit, and can facilitate the tissue regeneration in response to SCF stimulation. Activation of SCF/c-kit signaling in the progenitor cell niche stimulates several pathways mediating proliferation, survival, and migration (Stankov et al., 2014). We have previously identified a population of c-kit^+^ RPCs in retinas of postnatal mice. Both photoreceptors in the outer nuclear layer, and retinal neurons and Müller cells in the INL are the progeny of c-kit^+^ cells *in vivo* (Chen et al., 2017). Further, we have demonstrated that subretinal transplantation of c-kit^+^ cells, either isolated from newborn mice retinas or human embryonic stem cell-derived retinal organoids, can improve the visual function in retinal degeneration mice (Chen et al., 2016; Zou et al., 2019). Both of these findings suggest the protective role of c-kit^+^ cells in the degenerative retinal diseases. Intriguingly, we also found that c-kit expression persisted at low levels in retinas of adult mice, up to 57 weeks of age (Chen et al., 2017). However, whether SCF/c-kit signaling in the retina of adult mice can also facilitate the restoration of the retinal function remains unclear. In the present study, we found that in degenerative retinas, the proportion of c-kit^+^ cells increased, while the expression of c-kit ligand SCF decreased. After supplementing SCF exogenously, the NMDA-induced RGC loss was alleviated, and reductions in visual function after NMDA treatment were ameliorated, suggesting that the SCF/c-kit signaling contributes to the tissue homeostasis in the mice retinas. When the proliferating cells labeled by proliferating cell nuclear antigen (PCNA), we did not spot proliferating cells in GCL (c-kit^+^ cells or other RGCs). Very few of c-kit^+^ cells in INL were marked by PCNA, indicating low level of proliferating c-kit^+^ cells in INL. Above all, we speculated that SCF treatment might attenuate the loss of RGC, instead of c-kit^+^ cells proliferating and differentiating into new RGC. In the future research, tracing the changes of RGC through lineage tracing animal models may help us to better identify the origin of RGC.

Müller cells can interact with neurons, and are responsible for the maintenance of the homeostasis of the retina. In the present study, we found that the endogenous SCF was mainly derived from Müller cells. Intriguingly, we found that Cx43 was distributed between c-kit^+^ cells and SCF-expressing Müller cells, suggesting physical connection between these cells may exist. In the retina, Cx43 is expressed within the perivascular endfeet of astrocytes, Müller cells, retinal pigment epithelium and some neurons, and has been functionally associated with vascular regulation (Gonzalez-Casanova et al., 2021). The regulatory role of Cx43 in the endogenous SCF/c-kit signaling pathway has not been fully explored. Further investigation is needed to demonstrate the mechanisms and functional significances underlying these interactions.

Neuro-protective strategies could be promising to promote cell survival and prevent retinal neuron death (Pardue and Allen, 2018). Neuroprotective factors, including BDNF, ciliary neurotrophic factor, etc., have considerable potential to act as a powerful neuroprotective agent (Du et al., 2020; Dulz et al., 2020). However, significant challenges remain due to short half-lives of these factors and the inability to easily cross the blood brain or blood retina barrier (Pardue and Allen, 2018). Local delivery to the eye might avoid some of these limitations, while still causing increasing risk of infection and tissue injury. Here, we reported a long-lasting neuroprotective effect of SCF in the retinal degeneration mice. After a single administration of SCF, the protective effect sustained for at least 2 weeks, according to the reduction of RGC loss, the protection of RGC function and the restoration of visual function detected by light/dark transition test and fERG. The long-lasting effect of SCF has also been reported in the central nervous system (Qiu et al., 2020). In the traumatic brain injury (TBI) model, exogenous supplementation of SCF significantly showed superior efficacy in improving long-term functional outcome, enhancing neural plasticity, rebalancing neural structure networks disturbed by severe TBI, and promoting remyelination, as long as lasting for 21 weeks after treatment (Qiu et al., 2020). In addition, SCF has been covalently immobilized on polymeric substrate materials, such as hyaluronic acid/gelatin double network hydrogel, for the sustained release (Zhang et al., 2018). We will investigate the protective effect of these modifications of SCF in our further study.

During our study, Li et al. overexpressed SCF in photoreceptors by AAV8 virus, in attempts to treating photoreceptor degeneration (Li et al., 2020b). In support of our findings, this study indicated the role of SCF in preventing retinal degeneration. However, we and three independent groups all suggested that in the retina, the expression of c-kit is restricted to RGCs and amacrine cells, but not photoreceptors (Morii et al., 1994; Koso et al., 2007; Harada et al., 2011). Here our data reported the pro-survival role of SCF/c-kit signaling in NMDA-induced cytotoxicity in RGCs.

The fERG is a valuable tool for assessing the function of retinal cells and circuits *in vivo*. For very weak flashes from darkness, receptoral potential of opposite polarity to the b-wave dominates the ERG. This negative-going response has been called the scotopic threshold response (STR). The STR is thought to reflect activity of the proximal retinal, i.e. RGC and amacrine cell (Holcombe et al., 2008; Saszik et al., 2002). In mice, the STR comprises positive and negative elements (pSTR and nSTR). In our study, we found a significant increase in the pSTR of SCF-treated mice at 1- and 2-week post treatment (Figures 6A–C). Selective pSTR attenuation occurs following optic nerve crush or transection, suggesting this component is primarily of RGC origin (Bui and Fortune, 2004; Liu et al., 2014). Therefore, the differential effect of SCF treatment upon individual STR responses may reflect differential perturbation of RGC and amacrine cell function, which verified the therapeutic effects of SCF on RGCs. The photopic negative response (PhNR), a later negative going response evident in light adapted conditions, has been related to RGC activity in rodents. Besides, the decreased amplitude of PhNR is positively correlated with the loss of RGCs (Li et al., 2005; Chrysostomou and Crowston, 2013). Our study showed the PhNR amplitudes in the SCF-injected eyes were consistently increased (Figures 6D,E). Taken together, these data demonstrated that exogenous SCF supplementation protected RGCs from NMDA-induced cell death, therefore delayed the progression of the retinal degeneration.

NMDA has been described as the glutamate analog that shows the greatest potency in increasing calcium influx and inducing neurotoxicity. Previous results have shown that NMDA neurotoxicity induces significant changes in the full field ERG response, a thinning on the inner retinal layers, and RGC death. Electroretinogram results showed a significant decrease of both a-wave and b-wave amplitude in NMDA injuried mice (Zheng et al., 2015; Xiao et al., 2021). The RGCs are the final retinal elements in the direct pathway from the eye to the brain. The information gathered by photoreceptors is projected next onto bipolar cells, which, in turn, send projects to RGCs. With NMDA injury, the function of the RGCs is significantly hindered, which results in the transmission of light signal is blocked, and the overall function of the retina is reduced. Therefore, with SCF treatment attenuating the loss of RGCs, the amplitude of STR and PhNR of SCF treated NMDA mice was higher than NMDA mice without SCF treatment. As the function of RGCs was rescued, the functions of bipolar cells and photoreceptors were also protected to a certain extent. Therefore, the amplitudes of a-wave and b-wave in the SCF+NMDA group were higher than those in the NMDA injury group (Figure 3).

Microglia monitor and maintain the physiological homeostasis of their microenvironment. Previous studies have reported that SCF inhibits microglial proliferation *in vitro* when microglia are stimulated by CSF-1 (Zhang and Fedoroff, 1998). In our experiments, we showed that microglia was significantly activated after NMDA administration. With SCF treatment, the number of activated microglia were significantly decreased (Figures 4A,B). Microglia can acquire many morphological phenotypes. The “resting” microglia have a ramified shape and the “activated” microglia an ameboid shape. In the central nervous system, SCF-treated microglia showed a neuroprotective phenotype expressing anti-inflammatory cytokines, growth factors, and M2 markers as compared to the phenotype shown by granulocyte macrophage-colony stimulating factor-derived microglia expressing inflammatory cytokines and M1 markers (Terashima et al., 2018). Thus, we quantified the microglia morphology using a grid cross-counting system as reported previously (Luckoff et al., 2016; Luckoff et al., 2017; Bian et al., 2020). By counting the grid-crossed points of Iba1^+^ cells, we found that the microglia in the SCF-treated group mainly showed ramified shapes, while most microglia in the control group adopt an amoeboid morphology (Figure 4C). Taken together, these data demonstrated that exogenous SCF treatment can inhibit the hyperactivation of microglia in the NMDA-treated retina, and thus improve the visual function. Also, we found that c-kit was mainly expressed in neurons of GCL and INL, not in microglia in the retina (Figure 1). Therefore, we speculate that SCF could not directly act on retinal microglia, but reduce the activation of microglia by reducing neuron death and improving retinal microenvironment.

To further analyze the RNA-seq data, we found that the expression of multiple members from α, β and γ Crystallins family (*Cryaa*, *Cryab*, *Cryba1*, *Cryba2*, *Crygb*, *etc.*) were significantly up-regulated after SCF stimulation (Figure 7D), and also confirmed by real-time qPCR (Figure 7F). Both αA- and αB-crystallin has been reported to protect retinal neurons from cell death. Knockout of either αA- or αB-crystallin results in increased apoptosis and necroptosis of photoreceptors (Wang T. et al., 2021). Also, αB-Crystallin alleviates endotoxin-induced retinal inflammation and inhibits microglial activation and autophagy to protect injured retina (Wang F. et al., 2021). Our previous research also demonstrated that the Crystallins members plays an important role in promoting the survival of RGCs (A et al., 2019), indicating that the upregulation of Crystallins may be a critical downstream pathway in SCF-mediated retinal protection.

In addition, a series of factors that promote neuron survival were also significantly up-regulated with SCF exposure, including *Pitx3* and *Gja3* (Figures 7E,G), which are involved in neuronal degeneration, cell survival and immune modulation, and represent important candidate gene sets that regulate the retinal homeostasis (Anand et al., 2018; Akula et al., 2019; Li et al., 2020a). Also, it has been reported that these neuroprotective genes (including Gja3, Pitx3, Wnt7a, Foxe3, as well as Cryaa) are the top downregulated genes in the retinas of streptozotocin (STZ)-induced diabetic rats (Liu et al., 2016), suggesting this gene expressing profile may be characterized as a critical signature that play an important role in the maintenance of the retinal function. It has been documented that Wnt pathway is involved in processes of neurogenesis, dendritic development and axon guidance during development, and can inhibit neuronal damage by up-regulating anti-apoptotic proteins such as Survivin (Shi et al., 2020; Kassumeh et al., 2021). *In vitro*, exogenous administration of Wnt3a can bind to the frizzled receptor and LRP5/6 on the surface of RGCs, and promote the survival and axon regeneration of RGCs by down-regulating Ripk1 and Ripk3, *etc.* (Udeh et al., 2019). With NMDA-induced RGCs injury, Wnt pathway was significantly suppressed, suggesting that Wnt-related pathway may be critical for the survival of RGCs (Boesl et al., 2020). In the present study, we found the upon SCF stimulation, *Wnt7b* was significantly up-regulated, suggesting that this pathway may be critical for the protective effect of c-kit^+^ cells on RGCs (Figure 7G). Additional investigations, however, are needed to support this speculation, and both the origin and the targets in retina of Wnt7 should be addressed.

## Conclusion

In summary, our study demonstrates that in the NMDA-induced retinal degeneration mice model, endogenous c-kit^+^ cells can be stimulated by the treatment of SCF. The activation of c-kit^+^ cells confer protection against retinal degeneration, *via* inhibiting the loss of RGCs. Administration of SCF can act as a potent strategy for treating retinal degeneration-related diseases.

## Data Availability

The datasets presented in this study can be found in online repositories. The names of the repository/repositories and accession number(s) can be found below: GSE192458.
