# Supplementary figures and images for: Stimulation of C-Kit+ Retinal Progenitor Cells by Stem Cell Factor Confers Protection Against Retinal Degeneration
Source: Front Pharmacol. 2022 Mar 31;13:796380. doi: 10.3389/fphar.2022.796380 (PMC9008784; doi:10.3389/fphar.2022.796380)

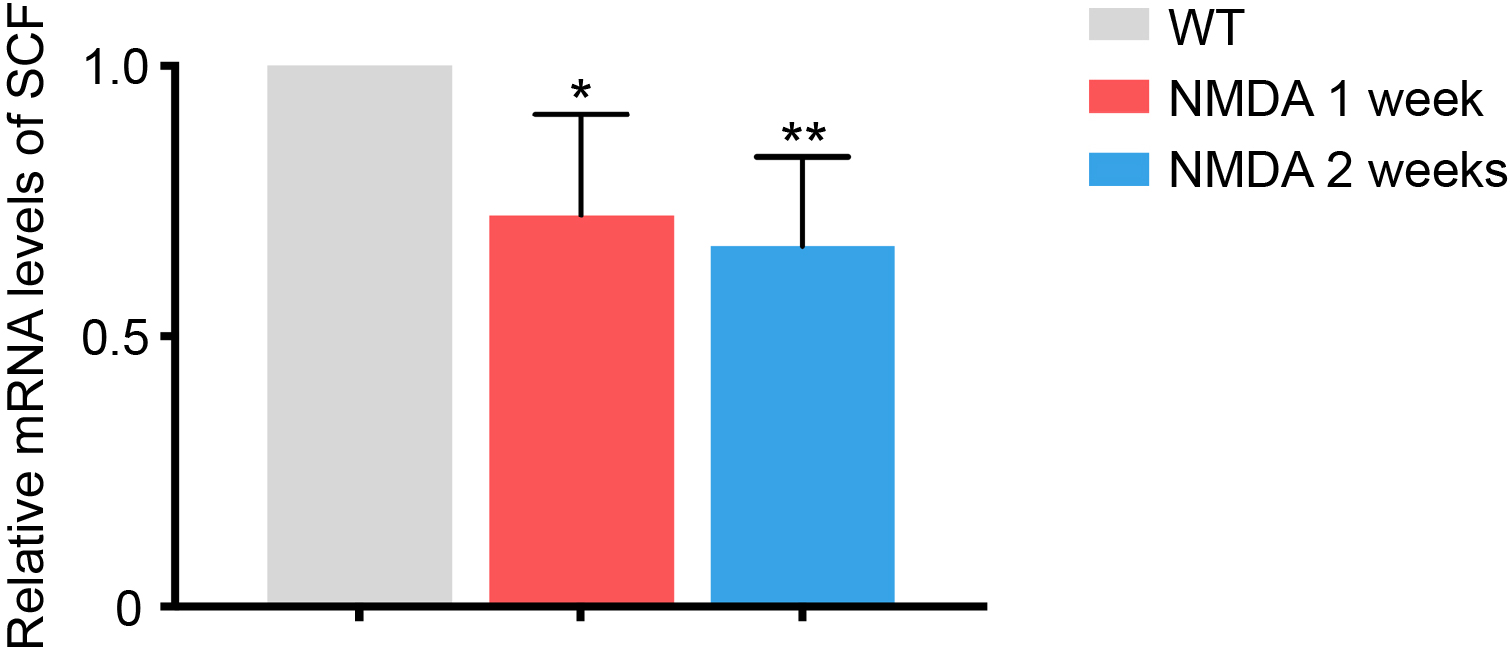

Supplement: Supplementary file 1 [file Image3.JPEG]

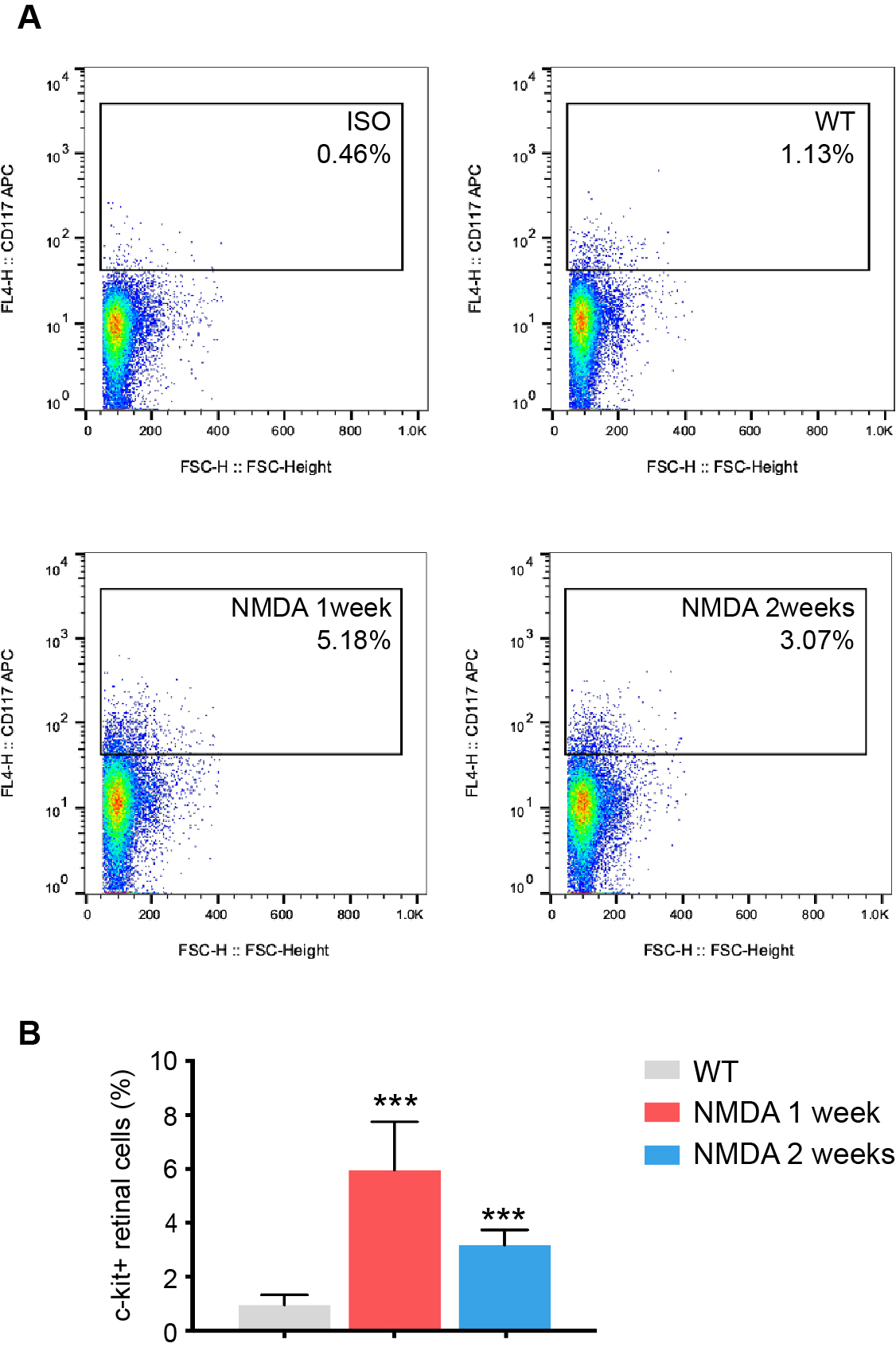

Supplement: Supplementary file 2 [file Image1.JPEG]

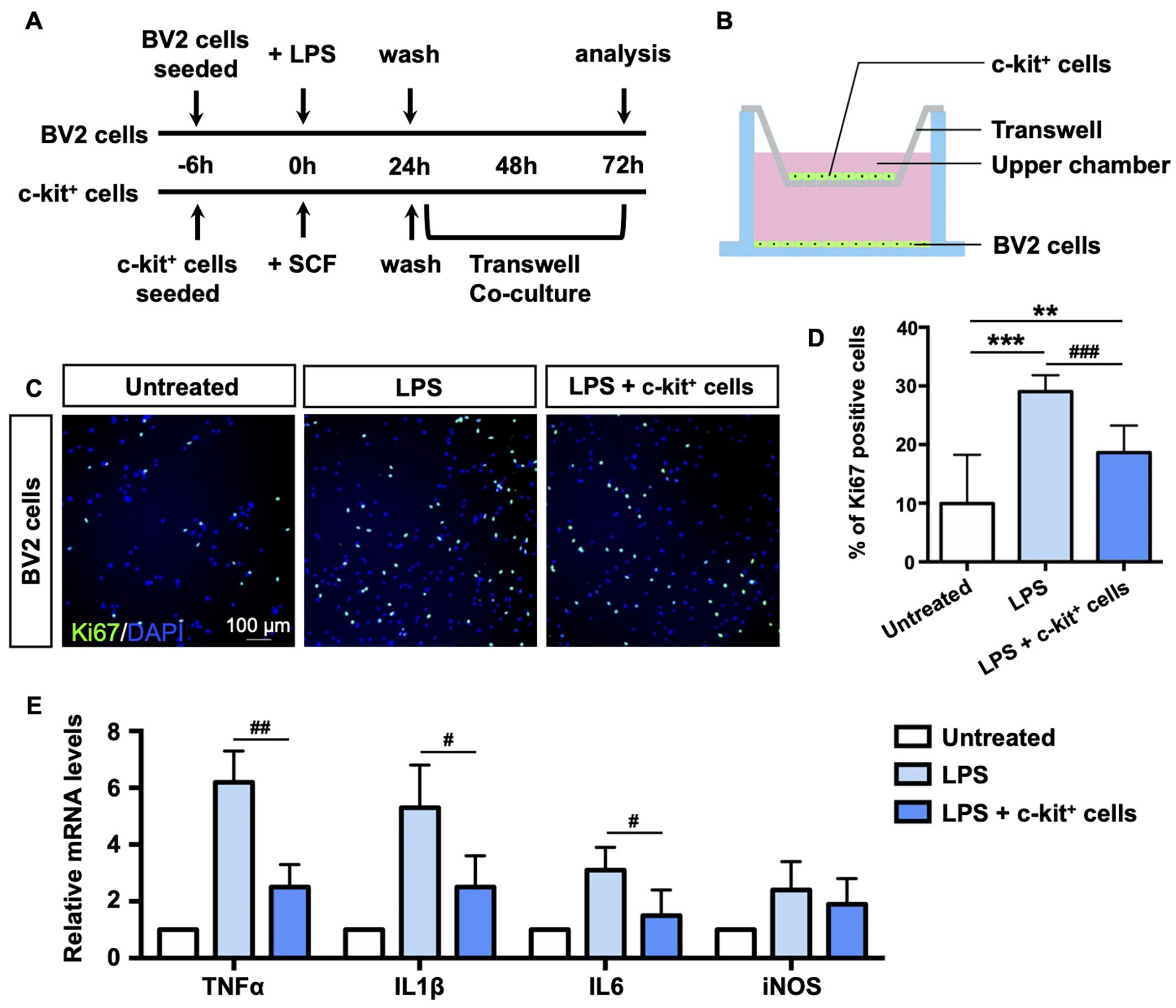

Supplement: Supplementary file 3 [file Image4.JPEG]

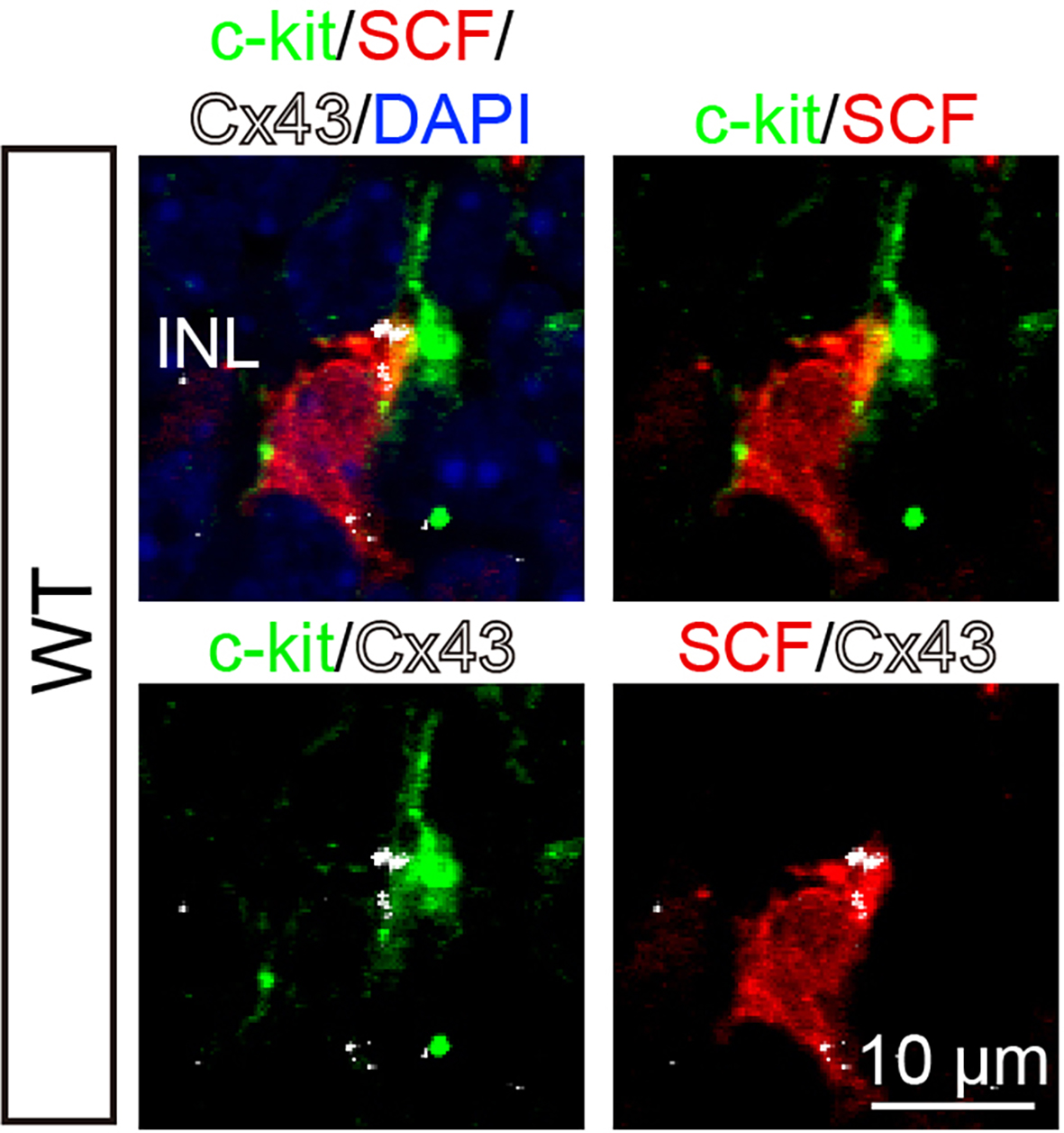

Supplement: Supplementary file 4 [file Image2.JPEG]

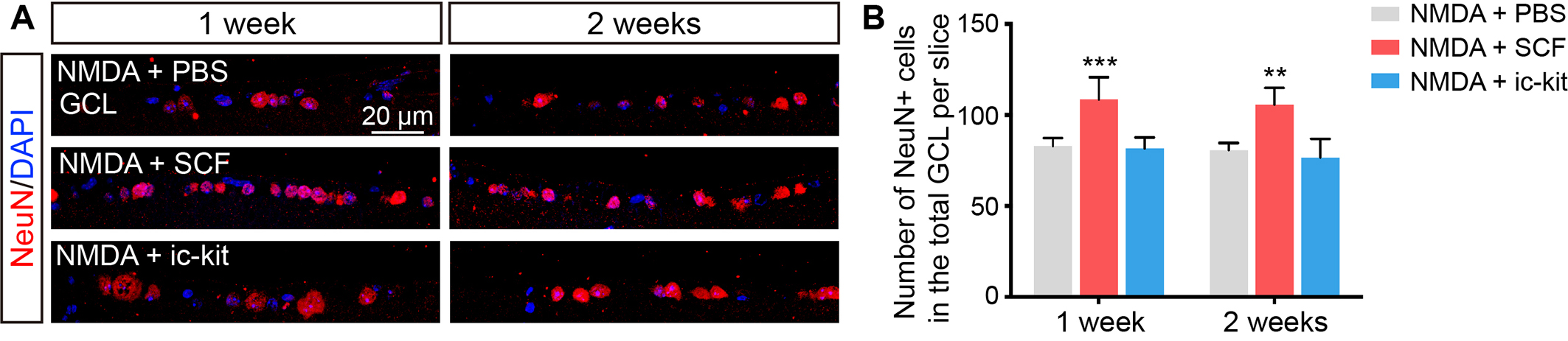

Supplement: Supplementary file 5 [file Image5.JPEG]

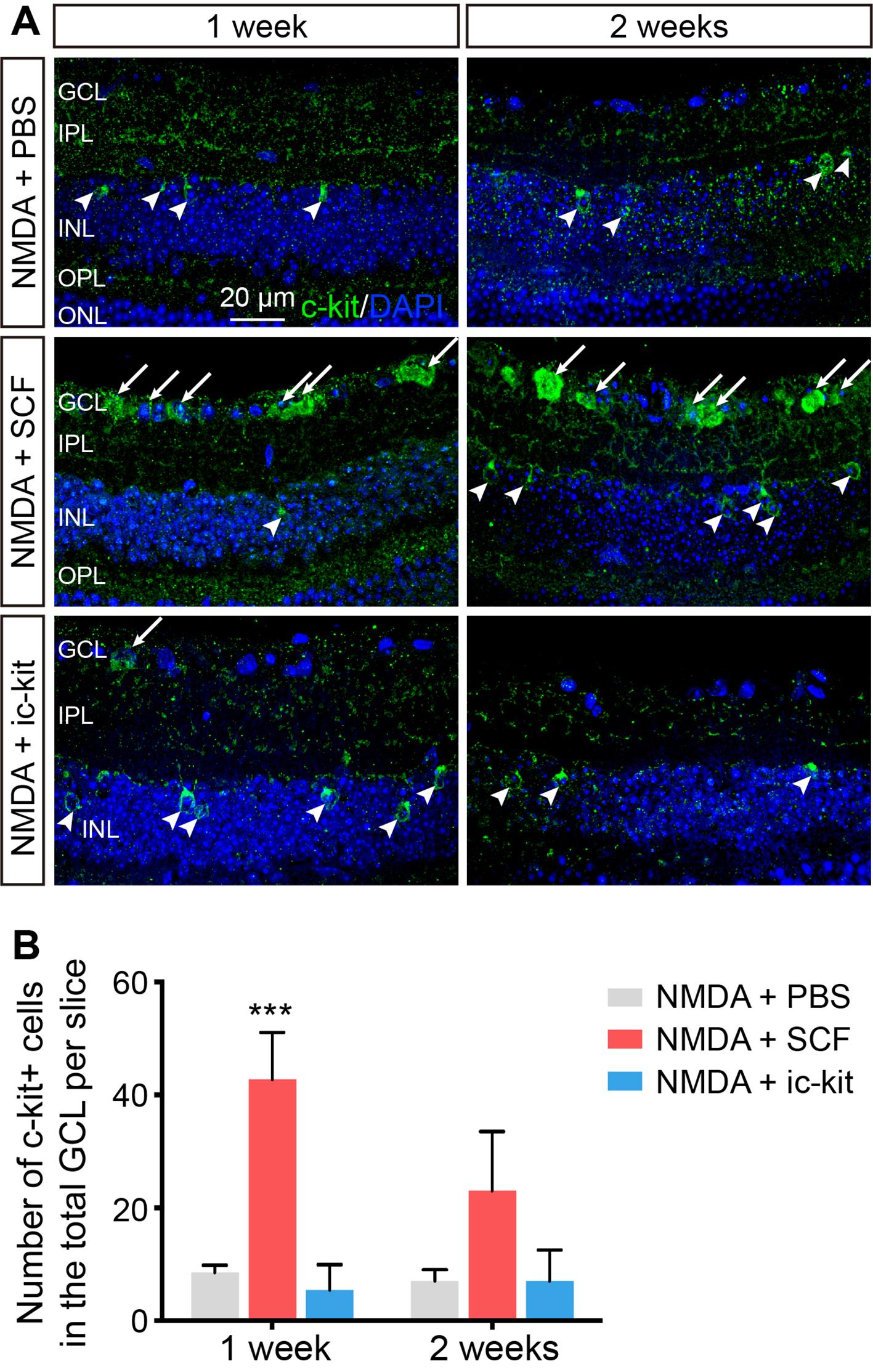

Supplement: Supplementary file 7 [file Image6.JPEG]
