# Supplementary material for: Stimulation of C-Kit+ Retinal Progenitor Cells by Stem Cell Factor Confers Protection Against Retinal Degeneration
Source: Front Pharmacol. 2022 Mar 31;13:796380. doi: 10.3389/fphar.2022.796380 (PMC9008784; doi:10.3389/fphar.2022.796380)
Supplement: Supplementary file 6 [file DataSheet1.docx]

Supplementary Material

Stimulation of C-kit^+^ Retinal Progenitor Cells by Stem Cell Factor Confers Protection against Retinal Degeneration

Xi Chen^1^*^†^, Shanshan Li^1†^, Xiaoli Liu^2^, Jingjie Zhao^3^, Lanting Wu^1^, Ran You^1^, Yanling Wang^1^*

^1^Department of Ophthalmology, Beijing Friendship Hospital, Capital Medical University, Beijing 100050, China

^2^Department of Pediatric Newborn Medicine, Brigham and Women’s Hospital and Harvard Medical School, Boston, Massachusetts 02115, United States

^3^Department of Traditional Chinese Medicine, Beijing Friendship Hospital, Capital Medical University, Beijing 100050, China

^†^Contributed equally to this study.

*** Correspondence:**

Xi Chen

xichen@ccmu.edu.cn

Yanling Wang
wangyanling999@vip.sina.com

***Supplementary Online Content List***

**Supplementary Figure S1.** Number of C-kit^+^ Cells in Wildtype (WT) and NMDA-treated Mice was Established by Flow Cytometry.

**Supplementary Figure S2.** Immunofluorescence detection showing Connexin 43 (Cx43) is located between c-kit^+^ cells and SCF^+^ cells in INL of retinas

**Supplementary Figure S3.** Real- time qPCR Analysis Showing Relative mRNA Expression for SCF between WT and NMDA Treated Mice.

**Supplementary Figure S4.** Stimulation of C-kit^+^ Cells by SCF Inhibited the Activation of BV2 Microglia Cells.

**Supplementary Figure S5.** RGCs was protected after SCF treatment against NMDA damage

**Supplementary Figure S6.** C-kit^+^ cells in NMDA injured mice treated with SCF and ic-kit after 1 and 2 weeks

**Supplementary Table S1.** Real-time qPCR Primers

**Supplementary Table S2.** GO enrichment analysis of the enriched pathways that are induced by SCF treatment


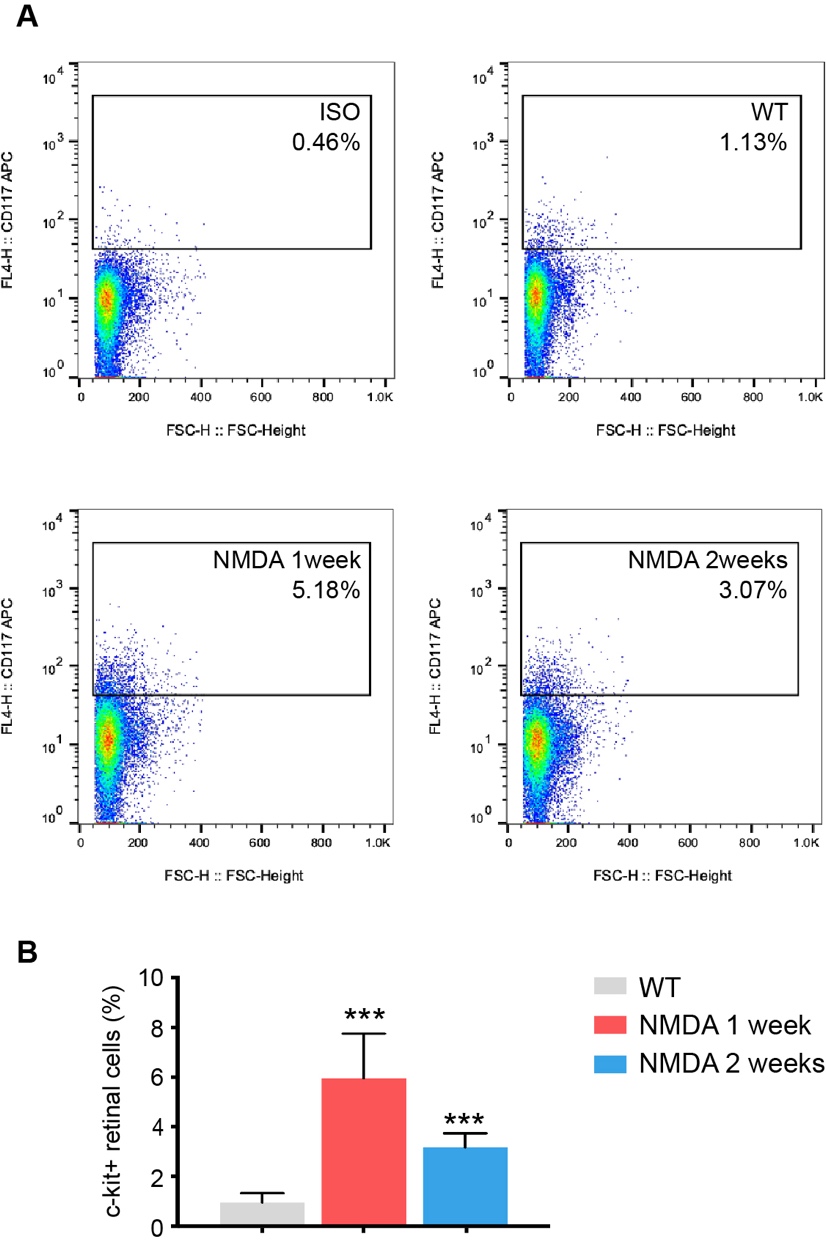


**Supplementary Figure S1.** Number of C-kit^+^ Cells in Wildtype (WT) and NMDA-treated Mice was Established by Flow Cytometry. (**A**) Flow cytometry assessment of c-kit^+^ (APC) cells as a % of total retina cells in wildtype (WT) mice and NMDA-treated mice. Representative flow cytometry scatter plots were showed at each time point. (**B**) Statistical analysis of the percentage of c-kit^+^ cells among groups. Data are shown as mean ± SD (n = 8 for each time point). *** *P* < 0.001 versus WT.


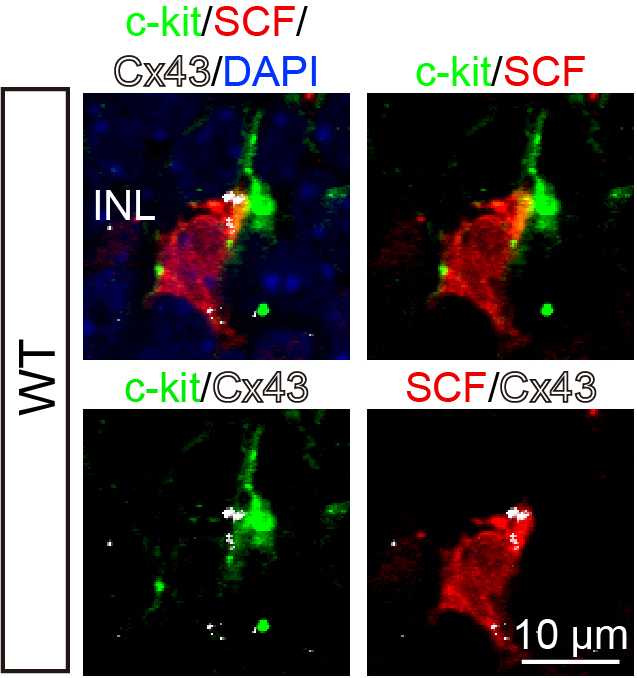


**Supplementary Figure S2.** Immunofluorescence detection showing Connexin 43 (Cx43) is located between c-kit^+^ cells and SCF^+^ cells in INL of retinas. Scale bars represent 10 μm.

**
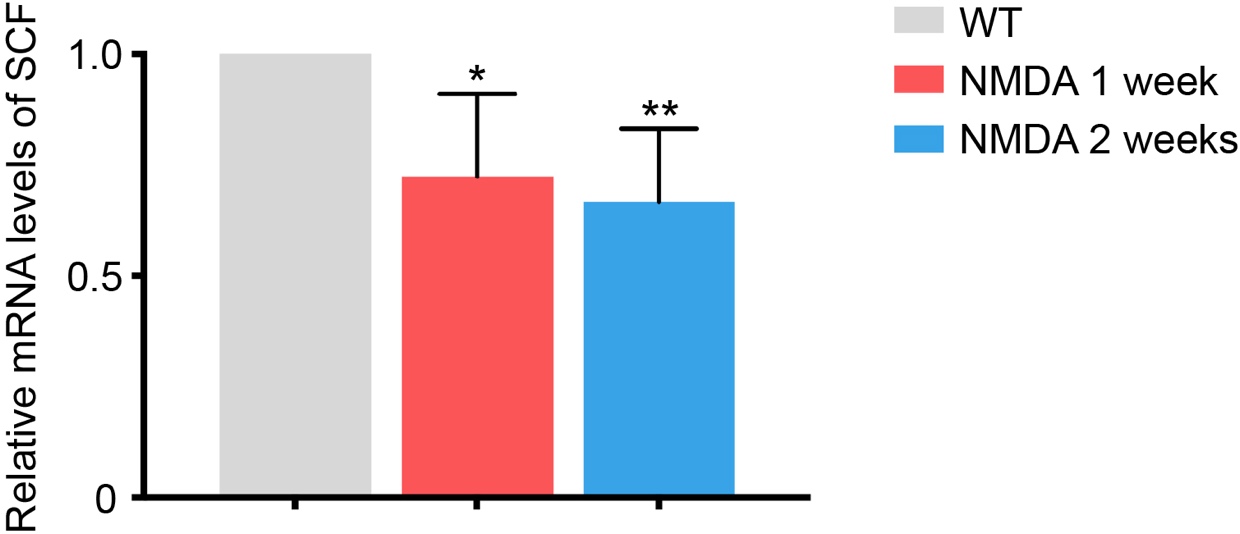
**

**Supplementary Figure S3. Real- time qPCR Analysis Showing Relative mRNA Expression for SCF between WT and NMDA Treated Mice.** Data are shown as mean ± SD (n = 5 for each time point). * *P* < 0.05 versus WT, ** *P* < 0.01 versus WT.

**
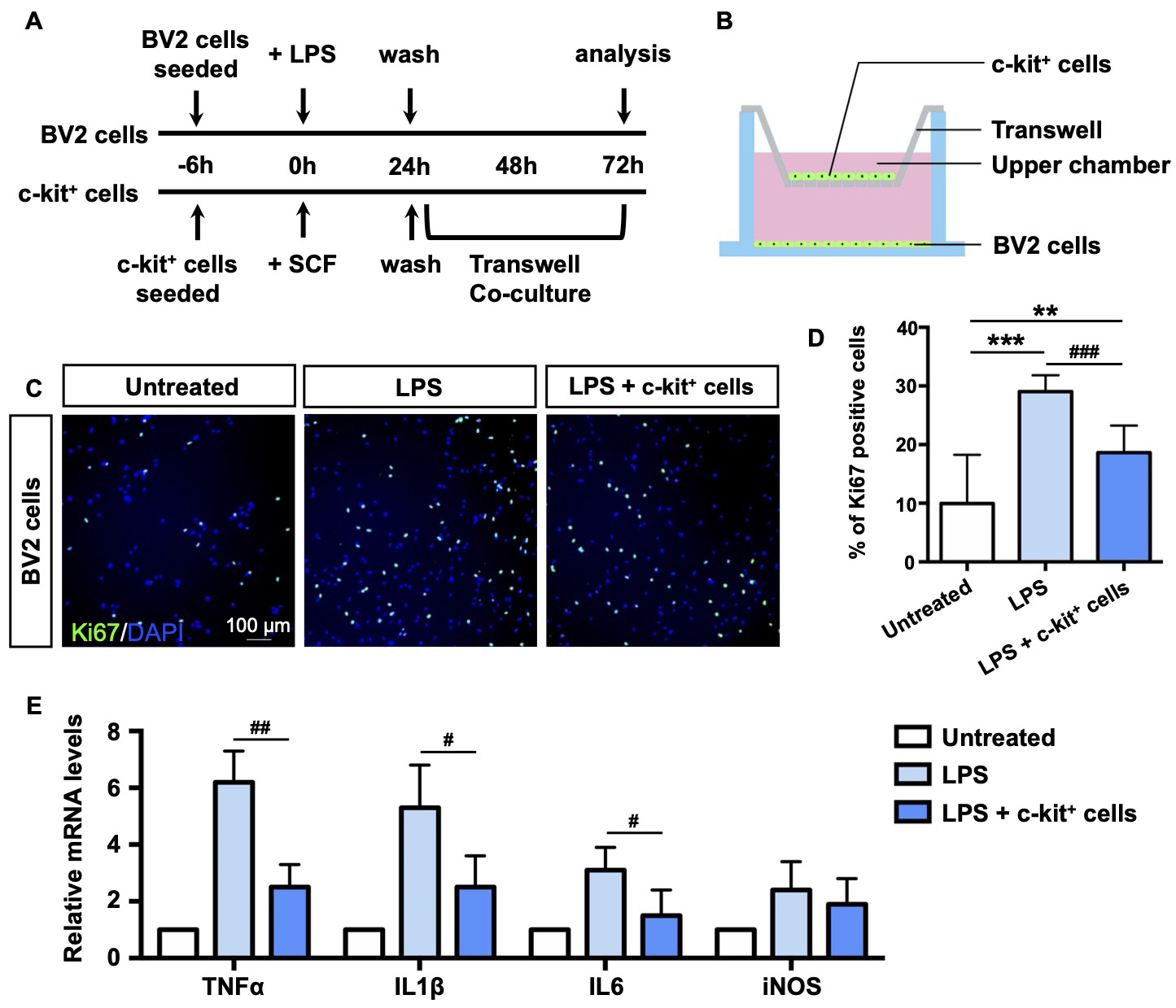
**

**Supplementary Figure S4. Stimulation of C-kit^+^ Cells by SCF Inhibited the Activation of BV2 Microglia Cells.** (**A**) Timetable of the co-culture system. LPS-activated BV2 cells and SCF-stimulated c-kit^+^ cells were cultured separately for 24 hours. After medium change, c-kit^+^ cells and BV2 cells were co-cultured for another 48 hours. (**B**) Diagram of co-culture system. (**C**) Representative images of immunofluorescence staining for Ki67 (green) with DAPI (blue) of indicated cells. (**D**) Statistical analysis of the ratio of Ki67-positive cells. (**E**) Real-time qPCR analysis of the expression of indicated genes. Data from at least three independent experiments are expressed as the mean ± SD. ***P* < 0.01, ****P* < 0.001, versus untreated BV2 cells; ^#^*P* < 0.05, ^##^*P* < 0.01, ^###^*P* < 0.001, between LPS activated BV2 cells with and without SCF stimulated c-kit^+^ cells.

**
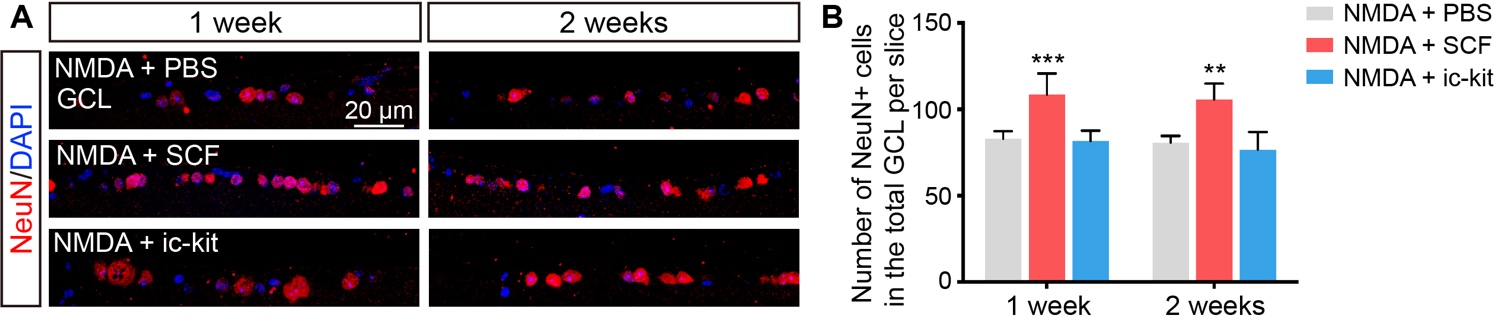
**

**Supplementary Figure S5.** RGCs was protected after SCF treatment against NMDA damage. (**A**) Immunofluorescence was used to detect the number of NeuN ^+^ RGCs (red) in the GCL of SCF group and ic-kit group for 1 and 2 weeks in retinas treated with NMDA. Scale bars represent 20 μm. (**B**) Statistical analysis of the number of NeuN ^+^ RGCs per slice among groups. Data are shown as mean ± SD (n = 5 for each time point). * *P* < 0.05, ** *P* < 0.01, compared with NMDA + PBS controls.


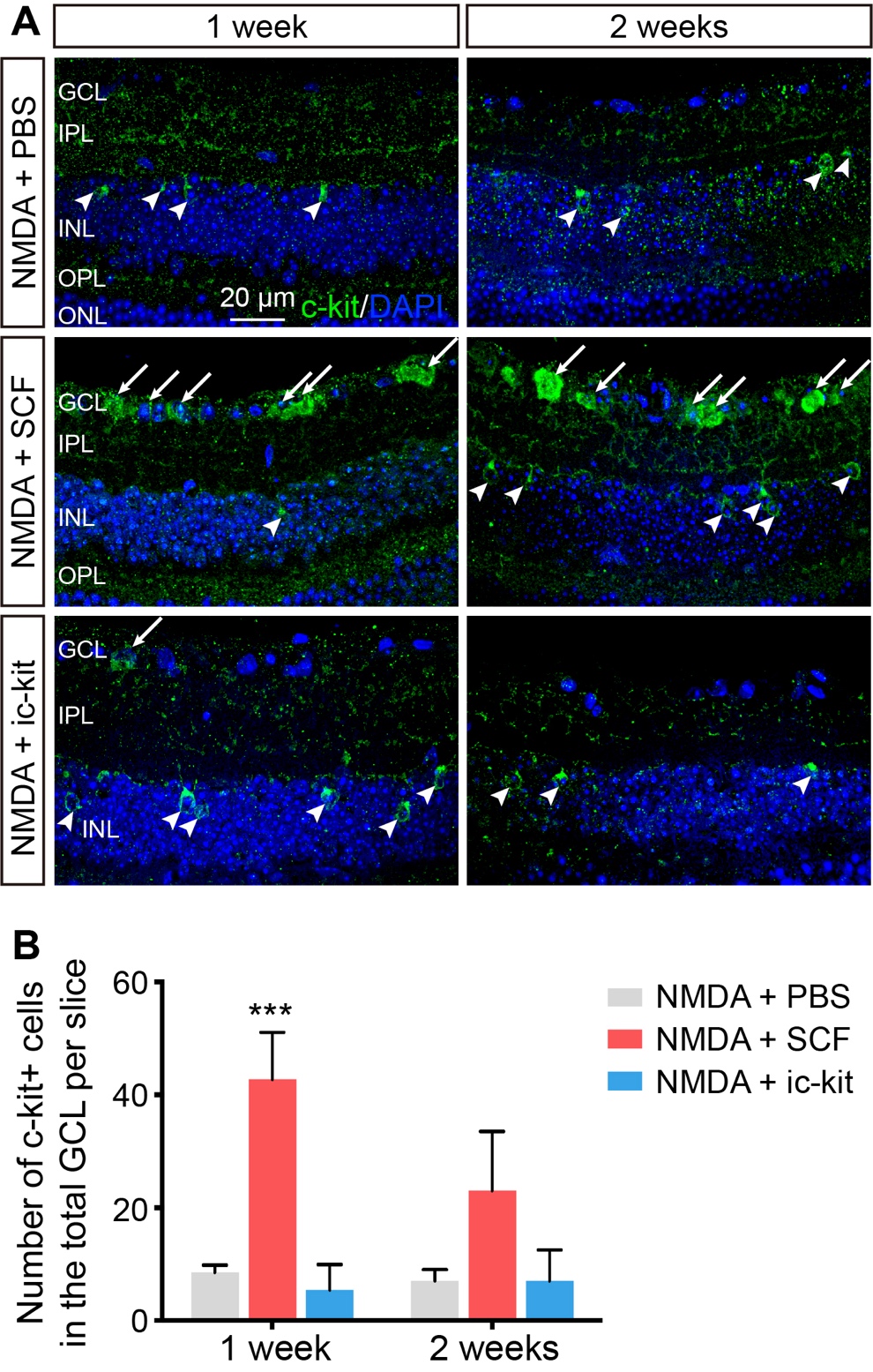


**Supplementary Figure S6.** C-kit^+^ cells in NMDA injured mice treated with SCF and ic-kit after 1 and 2 weeks. (**A**) Distribution and morphology of c-kit^+^ cells (green) in PBS control group (first row), SCF group (second row) and ic-kit group (third row) after corresponding treatment. Scale bars represent 20 μm. (**B**) Statistical analysis of the number of c-kit^+^ cells in the GCL among groups. White arrows point to the c-kit^+^ cell bodies in the GCL. IPL, inner plexiform layer, OPL, outer plexiform layer. Data are shown as mean ± SD (n = 5 for each time point). * *P* < 0.05, ** *P* < 0.01, *** *P* < 0.001, compared with control group.

**Supplementary Table S1. Real-time qPCR Primers**

| **Gene Symbol** | **Forward primer(5'-3')** | **Reverse primer** | **Product length (bp)** |
| --- | --- | --- | --- |
| *Pitx3* | GCCACCTTCCAGAGGAATC | TCTTGAACCACACCCGCA | 100 |
| *Foxe3* | TACCTGTGAGTCGCTTTCC | TACGGGTACCACGGACAA | 94 |
| *Gja3* | TTCCGCATTCTGGTGTTAGG | GGTCGTAGCAGACGTTCTC | 109 |
| *Gja8* | CAATGTGGTAGACTGCTTTGTA | TGACACAAAAGCGACTGATAAC | 82 |
| *Wnt7a* | GGACGCCATCATCGTCATAG | TCCAACGGCCATTTCGGAA | 80 |
| *Wnt7b* | TTCACCTATGCCATCACGG | GCCTGGTTGTAGTAGCCTT | 116 |
| *Rspo1* | TGCAAGATCGAGCACTGT | AGCGGCCCTTGTGTAAGTA | 85 |
| *Cryaa* | CCGGGACAAGTTTGTCATC | TCCACAAAATCCTCCAGTACC | 84 |
| *Cryab* | GAAGACTGCATATATAAGGGGC | GATGTCCATTGTGGCTAGATGA | 92 |
| *Cryba1* | TGGCTCAGACCAACCCTA | TCTTGCCCTGGAAGTTCTC | 81 |
| *Cryba2* | CTGTGCCAACGTCTGTGA | GAAGTCGGGGTACTCGAAG | 94 |
| *Cryba4* | CCGACGGCATGAATTCAC | TCAGGACTTTGAGAGATCGC | 80 |
| *Crygb* | CGATTGTCTCTCTCTTCAGGA | AGCTAGGCATCTCATAGAGAAC | 95 |
| *Crygs* | CGACTTCAACGGTCAGATG | GGAATGAATCTCTCGCAGG | 82 |
| *Crygn* | AGTGCCTAGAGTTCGTGG | CCATACACCTTGATGGCG | 85 |
| *Crybb1* | GACACCTGGACCAGCAGTTA | CTTCGAACAGGCAGATCTTATG | 94 |
| *Crybb2* | ACCTGAAGGAGACTGGTATG | GCAATTAGCCTGCTCGTAG | 83 |
| *Crybb3* | GAGCAGTTTGTTCTGGAGAAG | GACAGGAGAATGTCACTACG | 80 |
| *Crygc* | GCAGGTTCCCACAGAATG | TGGAAGCGATCCTGGATG | 98 |
| *SCF* | ATAGTGGATGACCTCGTGT | GGATCTAGTTTCTGGCCTCT | 84 |
| *GAPDH* | GCAAGGACACTGAGCAAGA | GGATGGAAATTGTGAGGGAG | 76 |

**Supplementary Table S2. GO enrichment analysis of the enriched pathways that are induced by SCF treatment**

| **#Term** | **Database** | **ID** | **Input number** | **Background number** | **All input number** | **All background number** | **Input frequency** | **Background frequency** | **P-Value** | **input_gene** | **Hyperlink_change** |
| --- | --- | --- | --- | --- | --- | --- | --- | --- | --- | --- | --- |
| structural constituent of eye lens | Molecular Function | GO:0005212 | 11 | 23 | 432 | 14773 | 0.02546296 | 0.00155689 | 1.1617E-11 | Crygn,214301:up\|Crybb2,12961:up\|Cryba2,12958:up\|Crygs,12970:up\|Lim2,233187:up\|Cryaa,12954:up\|Crybb3,12962:up\|Mip,17339:up\|Bfsp2,107993:up\|Crygb,12965:up\|Crygd,12967:up | http://amigo.geneontology.org/amigo/term/GO:0005212 |
| lens development in camera-type eye | Biological Process | GO:0002088 | 15 | 62 | 432 | 14773 | 0.03472222 | 0.00419685 | 2.0319E-10 | Sox1,20664:up\|Pitx3,18742:up\|Hipk2,15258:up\|Dlg1,13383:up\|Cryba2,12958:up\|Crygs,12970:up\|Lim2,233187:up\|Nf2,18016:up\|Cryaa,12954:up\|Mip,17339:up\|Bfsp2,107993:up\|Gja8,14616:up\|Foxe3,30923:up\|Crygb,12965:up\|Crygd,12967:up | http://amigo.geneontology.org/amigo/term/GO:0002088 |
| camera-type eye development | Biological Process | GO:0043010 | 25 | 270 | 432 | 14773 | 0.05787037 | 0.01827659 | 3.8007E-07 | Hdac2,15182:up\|Sox1,20664:up\|Dscam,13508:up\|Crybb2,12961:up\|Dlg1,13383:up\|Cryba2,12958:up\|Crygs,12970:up\|Lim2,233187:up\|Arid1a,93760:up\|Bfsp2,107993:up\|Foxe3,30923:up\|Slc25a25,227731:up\|Gpm6a,234267:up\|Pitx3,18742:up\|Chd7,320790:down\|Hipk2,15258:up\|Nf2,18016:up\|Jag1,16449:up\|Cryaa,12954:up\|Mip,17339:up\|Gja8,14616:up\|Tulp1,22157:down\|Cep290,216274:up\|Crygb,12965:up\|Crygd,12967:up | http://amigo.geneontology.org/amigo/term/GO:0043010 |
| eye development | Biological Process | GO:0001654 | 27 | 313 | 432 | 14773 | 0.0625 | 0.0211873 | 5.3561E-07 | Hdac2,15182:up\|Sox1,20664:up\|Dscam,13508:up\|Crybb2,12961:up\|Dlg1,13383:up\|Cryba2,12958:up\|Crygs,12970:up\|Lim2,233187:up\|Arid1a,93760:up\|Bfsp2,107993:up\|Foxe3,30923:up\|Slc25a25,227731:up\|Gpm6a,234267:up\|Pitx3,18742:up\|Chd7,320790:down\|Hipk2,15258:up\|Nf2,18016:up\|Jag1,16449:up\|Cryaa,12954:up\|Mip,17339:up\|Trpm1,17364:up\|Gja8,14616:up\|Tulp1,22157:down\|Prkci,18759:up\|Cep290,216274:up\|Crygb,12965:up\|Crygd,12967:up | http://amigo.geneontology.org/amigo/term/GO:0001654 |
| macromolecular complex subunit organization | Biological Process | GO:0043933 | 94 | 2000 | 432 | 14773 | 0.21759259 | 0.13538212 | 1.3863E-06 | Hdac2,15182:up\|Msl2,77853:up\|Ctr9,22083:up\|Tk1,21877:up\|Unc13b,22249:up\|Hist1h1e,50709:up\|Psmc6,67089:up\|Spc25,66442:up\|Fcho2,218503:up\|Napb,17957:up\|Rybp,56353:up\|Ndufaf4,68493:up\|Kdm3a,104263:up\|Pfkp,56421:up\|Clip1,56430:up\|Arfgef1,211673:up\|Kcna3,16491:up\|Cap2,67252:down\|Kctd6,71393:up\|Cd24a,12484:up\|Chd7,320790:down\|Atxn2,20239:up\|Cryaa,12954:up\|Chmp3,66700:up\|Wdr5,140858:up\|Mia3,338366:down\|Tet3,194388:down\|Enah,13800:up\|Cnot1,234594:up\|Kat6b,54169:up\|Elk4,13714:up\|Usp16,74112:up\|Dpysl3,22240:up\|Atrx,22589:up\|Bcl2l11,12125:up\|Gmfb,63985:up\|Sptbn1,20742:up\|Ptbp2,56195:up\|Kdm6a,22289:up\|Sept7,235072:up\|Nf2,18016:up\|Camk2d,108058:up\|Mip,17339:up\|Nap1l1,53605:up\|Gja8,14616:up\|Brcc3,210766:up\|Epb41,269587:up\|Nupl1,71844:up\|Capza2,12343:up\|Cbx6,494448:up\|Dlg1,13383:up\|Mgp,17313:down\|Dnm3,103967:up\|Wdr1,22388:up\|Emsy,233545:up\|Brd7,26992:down\|Fry,320365:up\|Ubr5,70790:up\|Actb,11461:down\|Eml1,68519:up\|Brd2,14312:down\|Arhgef5,54324:up\|Brd3,67382:up\|Rbbp4,19646:up\|Kdm1a,99982:up\|Huwe1,59026:up\|Cav2,12390:down\|Cadps,27062:up\|Gls,14660:up\|Birc2,11797:up\|Actl6a,56456:up\|Rps19,20085:down\|Eif4h,22384:up\|Tmod3,50875:up\|Setd2,235626:up\|Cdh1,12550:up\|Arid1a,93760:up\|Xrcc3,74335:up\|Irs1,16367:up\|Pfkl,18641:down\|Caly,68566:down\|Rnf20,109331:up\|Skp1a,21402:down\|Mbnl1,56758:up\|Tpm1,22003:up\|Col3a1,12825:down\|Trpm1,17364:up\|Trip12,14897:up\|Gatad2b,229542:up\|Baz1b,22385:up\|Tubg1,103733:down\|Mapk9,26420:up\|Prkci,18759:up\|Tet2,214133:up | http://amigo.geneontology.org/amigo/term/GO:0043933 |
| cellular component organization | Biological Process | GO:0016043 | 188 | 4840 | 432 | 14773 | 0.43518519 | 0.32762472 | 1.4082E-06 | Bbs9,319845:up\|Tk1,21877:up\|Unc13b,22249:up\|Hist1h1e,50709:up\|Psmc6,67089:up\|Spc25,66442:up\|Cep89,72140:up\|Deptor,97998:up\|Crmp1,12933:up\|Fcho2,218503:up\|Napb,17957:up\|Rybp,56353:up\|Bfsp2,107993:up\|Ndufaf4,68493:up\|Usp33,170822:up\|Mib1,225164:up\|Pfkp,56421:up\|Mpp5,56217:up\|Clip1,56430:up\|Kcna3,16491:up\|Rspo1,192199:up\|Cd24a,12484:up\|Elmo2,140579:down\|Psen1,19164:up\|Chd7,320790:down\|Atxn2,20239:up\|Mff,75734:up\|Chmp3,66700:up\|Wdr5,140858:up\|Mia3,338366:down\|Wnt7a,22421:up\|Yme1l1,27377:up\|Kat6b,54169:up\|Nup88,19069:up\|Bhlhb9,70237:up\|Tsg101,22088:down\|Dpysl2,12934:up\|Atrx,22589:up\|Gmfb,63985:up\|Fbxw7,50754:up\|Uso1,56041:up\|Vcl,22330:up\|Nudt16,75686:down\|Sptbn1,20742:up\|Crim1,50766:up\|Ptbp2,56195:up\|Kdm6a,22289:up\|Sept7,235072:up\|Camk2d,108058:up\|Prdx6,11758:up\|Mip,17339:up\|Ogn,18295:up\|Nap1l1,53605:up\|Gja8,14616:up\|Phgdh,236539:up\|Epb41,269587:up\|Eif4g2,13690:up\|Capza2,12343:up\|Cbx6,494448:up\|Dlg1,13383:up\|Abca4,11304:up\|Tapt1,231225:up\|Wdr1,22388:up\|Chn1,108699:up\|Ndufs1,227197:up\|Emsy,233545:up\|Map4k4,26921:up\|Ubr5,70790:up\|Tmem88,67020:down\|Kdm1a,99982:up\|Coro6,216961:down\|Ppp2r5a,226849:up\|Rhot2,214952:down\|Huwe1,59026:up\|Cav2,12390:down\|Mbtps1,56453:up\|Ap3b1,11774:up\|Gls,14660:up\|Birc2,11797:up\|Dock2,94176:down\|Rps19,20085:down\|Plxnb1,235611:up\|Cdh1,12550:up\|Plxna3,18846:up\|Map3k4,26407:up\|Xrcc3,74335:up\|Pfkl,18641:down\|Lin7a,108030:up\|Rnf20,109331:up\|Clcn3,12725:up\|Sema6c,20360:down\|Msh3,17686:down\|Mbnl1,56758:up\|Map2,17756:up\|Col3a1,12825:down\|Zdhhc22,238331:up\|Anapc5,59008:up\|Gatad2b,229542:up\|Baz1b,22385:up\|Lims2,225341:down\|Tubg1,103733:down\|Prkci,18759:up\|Msl2,77853:up\|Hdac2,15182:up\|Gdap1,14545:up\|Plxna2,18845:up\|Ctr9,22083:up\|Ift74,67694:up\|Trp53inp2,68728:up\|Cacna1a,12286:up\|Kdm3a,104263:up\|Arfgef1,211673:up\|Cap2,67252:down\|Kctd6,71393:up\|Gpm6a,234267:up\|Cryaa,12954:up\|Tet3,194388:down\|Csnk1g1,214897:up\|Ncapg2,76044:up\|Enah,13800:up\|Kif3c,16570:up\|Crygb,12965:up\|Cep290,216274:up\|Ccng1,12450:up\|Cnot1,234594:up\|Elk4,13714:up\|Syt10,54526:up\|Usp16,74112:up\|Dscam,13508:up\|Dpysl3,22240:up\|Tulp2,56734:down\|U2af2,22185:down\|Hnrnpa2b1,53379:up\|Ptprf,19268:up\|Cxcl12,20315:up\|Bcl2l11,12125:up\|Ero1l,50527:up\|Lyst,17101:up\|Sfrp1,20377:up\|Nid1,18073:up\|Nf2,18016:up\|Tmem237,381259:up\|Epb41l2,13822:up\|Lmtk2,231876:up\|Brcc3,210766:up\|Nupl1,71844:up\|Ccdc64,75665:down\|Mgp,17313:down\|Dnm3,103967:up\|Aplp2,11804:up\|Brd7,26992:down\|Fry,320365:up\|Actb,11461:down\|Col4a4,12829:up\|Eml1,68519:up\|Brd2,14312:down\|Arhgef5,54324:up\|Brd3,67382:up\|Rbbp4,19646:up\|Ankfy1,11736:up\|Cadps,27062:up\|Rb1cc1,12421:up\|Tulp1,22157:down\|Actl6a,56456:up\|Mtif2,76784:down\|Eif4h,22384:up\|Tmod3,50875:up\|Setd2,235626:up\|Lix1,66643:up\|Marf1,223989:up\|Arid1a,93760:up\|Nme6,54369:down\|Nedd4,17999:up\|Irs1,16367:up\|Caly,68566:down\|Wee1,22390:up\|Dclre1c,227525:up\|Skp1a,21402:down\|Cfap20,14894:up\|Derl2,116891:up\|Ube2d3,66105:up\|Tpm1,22003:up\|Col4a3,12828:up\|Trpm1,17364:up\|Trip12,14897:up\|Tacc1,320165:down\|Mapk9,26420:up\|Tet2,214133:up | http://amigo.geneontology.org/amigo/term/GO:0016043 |
| covalent chromatin modification | Biological Process | GO:0016569 | 32 | 434 | 432 | 14773 | 0.07407407 | 0.02937792 | 1.5813E-06 | Hdac2,15182:up\|Msl2,77853:up\|Ctr9,22083:up\|Cbx6,494448:up\|Hist1h1e,50709:up\|Emsy,233545:up\|Rybp,56353:up\|Brd7,26992:down\|Kdm3a,104263:up\|Ubr5,70790:up\|Brd2,14312:down\|Brd3,67382:up\|Rbbp4,19646:up\|Kdm1a,99982:up\|Chd7,320790:down\|Huwe1,59026:up\|Wdr5,140858:up\|Tet3,194388:down\|Actl6a,56456:up\|Elk4,13714:up\|Kat6b,54169:up\|Setd2,235626:up\|Usp16,74112:up\|Atrx,22589:up\|Arid1a,93760:up\|Rnf20,109331:up\|Skp1a,21402:down\|Kdm6a,22289:up\|Trip12,14897:up\|Baz1b,22385:up\|Tet2,214133:up\|Brcc3,210766:up | http://amigo.geneontology.org/amigo/term/GO:0016569 |
| chromatin modification | Biological Process | GO:0016568 | 34 | 479 | 432 | 14773 | 0.0787037 | 0.03242402 | 1.7445E-06 | Hdac2,15182:up\|Msl2,77853:up\|Ctr9,22083:up\|Cbx6,494448:up\|Hist1h1e,50709:up\|Emsy,233545:up\|Rybp,56353:up\|Brd7,26992:down\|Kdm3a,104263:up\|Ubr5,70790:up\|Actb,11461:down\|Brd2,14312:down\|Brd3,67382:up\|Rbbp4,19646:up\|Kdm1a,99982:up\|Chd7,320790:down\|Huwe1,59026:up\|Wdr5,140858:up\|Tet3,194388:down\|Actl6a,56456:up\|Setd2,235626:up\|Elk4,13714:up\|Kat6b,54169:up\|Usp16,74112:up\|Arid1a,93760:up\|Atrx,22589:up\|Rnf20,109331:up\|Skp1a,21402:down\|Kdm6a,22289:up\|Gatad2b,229542:up\|Trip12,14897:up\|Baz1b,22385:up\|Tet2,214133:up\|Brcc3,210766:up | http://amigo.geneontology.org/amigo/term/GO:0016568 |
| regulation of histone ubiquitination | Biological Process | GO:0033182 | 5 | 9 | 432 | 14773 | 0.01157407 | 0.00060922 | 2.3896E-06 | Kdm1a,99982:up\|Trip12,14897:up\|Ctr9,22083:up\|Ubr5,70790:up\|Rnf20,109331:up | http://amigo.geneontology.org/amigo/term/GO:0033182 |
| organelle organization | Biological Process | GO:0006996 | 126 | 2970 | 432 | 14773 | 0.29166667 | 0.20104244 | 3.2421E-06 | Hdac2,15182:up\|Msl2,77853:up\|Ctr9,22083:up\|Bbs9,319845:up\|Gdap1,14545:up\|Hist1h1e,50709:up\|Spc25,66442:up\|Cep89,72140:up\|Ift74,67694:up\|Crmp1,12933:up\|Fcho2,218503:up\|Trp53inp2,68728:up\|Rybp,56353:up\|Bfsp2,107993:up\|Ndufaf4,68493:up\|Kdm3a,104263:up\|Usp33,170822:up\|Pfkp,56421:up\|Clip1,56430:up\|Arfgef1,211673:up\|Cap2,67252:down\|Rspo1,192199:up\|Cd24a,12484:up\|Elmo2,140579:down\|Psen1,19164:up\|Chd7,320790:down\|Atxn2,20239:up\|Cryaa,12954:up\|Mff,75734:up\|Chmp3,66700:up\|Wdr5,140858:up\|Mia3,338366:down\|Tet3,194388:down\|Ncapg2,76044:up\|Enah,13800:up\|Cep290,216274:up\|Yme1l1,27377:up\|Ccng1,12450:up\|Cnot1,234594:up\|Kat6b,54169:up\|Elk4,13714:up\|Nup88,19069:up\|Usp16,74112:up\|Syt10,54526:up\|Tsg101,22088:down\|Dpysl3,22240:up\|Dpysl2,12934:up\|Atrx,22589:up\|U2af2,22185:down\|Hnrnpa2b1,53379:up\|Bcl2l11,12125:up\|Gmfb,63985:up\|Fbxw7,50754:up\|Uso1,56041:up\|Lyst,17101:up\|Nudt16,75686:down\|Sptbn1,20742:up\|Sfrp1,20377:up\|Kdm6a,22289:up\|Sept7,235072:up\|Nf2,18016:up\|Tmem237,381259:up\|Epb41l2,13822:up\|Nap1l1,53605:up\|Epb41,269587:up\|Brcc3,210766:up\|Capza2,12343:up\|Cbx6,494448:up\|Dlg1,13383:up\|Dnm3,103967:up\|Tapt1,231225:up\|Wdr1,22388:up\|Ndufs1,227197:up\|Emsy,233545:up\|Fry,320365:up\|Brd7,26992:down\|Ubr5,70790:up\|Actb,11461:down\|Eml1,68519:up\|Brd2,14312:down\|Arhgef5,54324:up\|Brd3,67382:up\|Rbbp4,19646:up\|Kdm1a,99982:up\|Coro6,216961:down\|Ankfy1,11736:up\|Rhot2,214952:down\|Huwe1,59026:up\|Cav2,12390:down\|Mbtps1,56453:up\|Ap3b1,11774:up\|Cadps,27062:up\|Rb1cc1,12421:up\|Dock2,94176:down\|Actl6a,56456:up\|Rps19,20085:down\|Mtif2,76784:down\|Plxnb1,235611:up\|Tmod3,50875:up\|Setd2,235626:up\|Lix1,66643:up\|Marf1,223989:up\|Plxna3,18846:up\|Arid1a,93760:up\|Nme6,54369:down\|Map3k4,26407:up\|Xrcc3,74335:up\|Caly,68566:down\|Wee1,22390:up\|Rnf20,109331:up\|Dclre1c,227525:up\|Clcn3,12725:up\|Skp1a,21402:down\|Msh3,17686:down\|Map2,17756:up\|Ube2d3,66105:up\|Tpm1,22003:up\|Trip12,14897:up\|Anapc5,59008:up\|Gatad2b,229542:up\|Baz1b,22385:up\|Tacc1,320165:down\|Tubg1,103733:down\|Mapk9,26420:up\|Prkci,18759:up\|Tet2,214133:up | http://amigo.geneontology.org/amigo/term/GO:0006996 |
| protein modification by small protein conjugation or removal | Biological Process | GO:0070647 | 41 | 654 | 432 | 14773 | 0.09490741 | 0.04426995 | 3.5396E-06 | Ctr9,22083:up\|Wwp2,66894:up\|Rffl,67338:up\|Rybp,56353:up\|Ccnc,51813:up\|Ubr5,70790:up\|Usp33,170822:up\|Mib1,225164:up\|Tank,21353:up\|Ufm1,67890:up\|Kdm1a,99982:up\|Ahrr,11624:up\|Nub1,53312:up\|Psen1,19164:up\|Huwe1,59026:up\|Usp12,22217:up\|Fbxl5,242960:up\|Senp3,80886:up\|Klhl11,217194:up\|Birc2,11797:up\|Usp7,252870:up\|Usp16,74112:up\|Arnt,11863:up\|Tsg101,22088:down\|Cdk8,264064:up\|Nedd4,17999:up\|Xrcc3,74335:up\|Fbxw7,50754:up\|Rnf20,109331:up\|Usp19,71472:up\|N4bp1,80750:up\|Skp1a,21402:down\|Rnf111,93836:up\|Ube2d3,66105:up\|Fbxl3,50789:up\|Anapc5,59008:up\|Trip12,14897:up\|Mapk9,26420:up\|Usp48,170707:up\|Rnf122,68867:down\|Brcc3,210766:up | http://amigo.geneontology.org/amigo/term/GO:0070647 |
| cellular component organization or biogenesis | Biological Process | GO:0071840 | 190 | 4982 | 432 | 14773 | 0.43981481 | 0.33723685 | 4.4397E-06 | Bbs9,319845:up\|Tk1,21877:up\|Unc13b,22249:up\|Hist1h1e,50709:up\|Psmc6,67089:up\|Spc25,66442:up\|Cep89,72140:up\|Deptor,97998:up\|Crmp1,12933:up\|Fcho2,218503:up\|Napb,17957:up\|Rybp,56353:up\|Bfsp2,107993:up\|Ndufaf4,68493:up\|Usp33,170822:up\|Mib1,225164:up\|Pfkp,56421:up\|Mpp5,56217:up\|Clip1,56430:up\|Kcna3,16491:up\|Rspo1,192199:up\|Cd24a,12484:up\|Elmo2,140579:down\|Psen1,19164:up\|Chd7,320790:down\|Atxn2,20239:up\|Mff,75734:up\|Chmp3,66700:up\|Wdr5,140858:up\|Mia3,338366:down\|Wnt7a,22421:up\|Yme1l1,27377:up\|Kat6b,54169:up\|Nup88,19069:up\|Bhlhb9,70237:up\|Tsg101,22088:down\|Dpysl2,12934:up\|Atrx,22589:up\|Gmfb,63985:up\|Fbxw7,50754:up\|Uso1,56041:up\|Vcl,22330:up\|Nudt16,75686:down\|Sptbn1,20742:up\|Crim1,50766:up\|Ptbp2,56195:up\|Kdm6a,22289:up\|Sept7,235072:up\|Camk2d,108058:up\|Prdx6,11758:up\|Mip,17339:up\|Ogn,18295:up\|Nap1l1,53605:up\|Gja8,14616:up\|Phgdh,236539:up\|Epb41,269587:up\|Eif4g2,13690:up\|Capza2,12343:up\|Cbx6,494448:up\|Dlg1,13383:up\|Abca4,11304:up\|Tapt1,231225:up\|Wdr1,22388:up\|Chn1,108699:up\|Ndufs1,227197:up\|Emsy,233545:up\|Map4k4,26921:up\|Ubr5,70790:up\|Tmem88,67020:down\|Kdm1a,99982:up\|Coro6,216961:down\|Ppp2r5a,226849:up\|Rhot2,214952:down\|Huwe1,59026:up\|Cav2,12390:down\|Mbtps1,56453:up\|Ap3b1,11774:up\|Gls,14660:up\|Birc2,11797:up\|Dock2,94176:down\|Rps19,20085:down\|Plxnb1,235611:up\|Cdh1,12550:up\|Plxna3,18846:up\|Map3k4,26407:up\|Xrcc3,74335:up\|Pfkl,18641:down\|Lin7a,108030:up\|Rnf20,109331:up\|Clcn3,12725:up\|Sema6c,20360:down\|Msh3,17686:down\|Mbnl1,56758:up\|Map2,17756:up\|Col3a1,12825:down\|Zdhhc22,238331:up\|Anapc5,59008:up\|Gatad2b,229542:up\|Baz1b,22385:up\|Lims2,225341:down\|Tubg1,103733:down\|Prkci,18759:up\|Msl2,77853:up\|Hdac2,15182:up\|Gdap1,14545:up\|Plxna2,18845:up\|Ctr9,22083:up\|Ift74,67694:up\|Trp53inp2,68728:up\|Cacna1a,12286:up\|Mettl16,67493:up\|Kdm3a,104263:up\|Arfgef1,211673:up\|Cap2,67252:down\|Kctd6,71393:up\|Gpm6a,234267:up\|Cryaa,12954:up\|Tet3,194388:down\|Csnk1g1,214897:up\|Ncapg2,76044:up\|Nol11,68979:up\|Enah,13800:up\|Kif3c,16570:up\|Crygb,12965:up\|Cep290,216274:up\|Ccng1,12450:up\|Cnot1,234594:up\|Elk4,13714:up\|Syt10,54526:up\|Usp16,74112:up\|Dscam,13508:up\|Dpysl3,22240:up\|Tulp2,56734:down\|U2af2,22185:down\|Hnrnpa2b1,53379:up\|Ptprf,19268:up\|Cxcl12,20315:up\|Bcl2l11,12125:up\|Ero1l,50527:up\|Lyst,17101:up\|Sfrp1,20377:up\|Nid1,18073:up\|Nf2,18016:up\|Tmem237,381259:up\|Epb41l2,13822:up\|Lmtk2,231876:up\|Brcc3,210766:up\|Nupl1,71844:up\|Ccdc64,75665:down\|Mgp,17313:down\|Dnm3,103967:up\|Aplp2,11804:up\|Brd7,26992:down\|Fry,320365:up\|Actb,11461:down\|Col4a4,12829:up\|Eml1,68519:up\|Brd2,14312:down\|Arhgef5,54324:up\|Brd3,67382:up\|Rbbp4,19646:up\|Ankfy1,11736:up\|Cadps,27062:up\|Rb1cc1,12421:up\|Tulp1,22157:down\|Actl6a,56456:up\|Mtif2,76784:down\|Eif4h,22384:up\|Tmod3,50875:up\|Setd2,235626:up\|Lix1,66643:up\|Marf1,223989:up\|Arid1a,93760:up\|Nme6,54369:down\|Nedd4,17999:up\|Irs1,16367:up\|Caly,68566:down\|Wee1,22390:up\|Dclre1c,227525:up\|Skp1a,21402:down\|Cfap20,14894:up\|Derl2,116891:up\|Ube2d3,66105:up\|Tpm1,22003:up\|Col4a3,12828:up\|Trpm1,17364:up\|Trip12,14897:up\|Tacc1,320165:down\|Mapk9,26420:up\|Tet2,214133:up | http://amigo.geneontology.org/amigo/term/GO:0071840 |
| lens fiber cell differentiation | Biological Process | GO:0070306 | 7 | 26 | 432 | 14773 | 0.0162037 | 0.00175997 | 7.0765E-06 | Bfsp2,107993:up\|Pitx3,18742:up\|Foxe3,30923:up\|Nf2,18016:up\|Crygb,12965:up\|Cryaa,12954:up\|Crygd,12967:up | http://amigo.geneontology.org/amigo/term/GO:0070306 |
| chromatin organization | Biological Process | GO:0006325 | 35 | 538 | 432 | 14773 | 0.08101852 | 0.03641779 | 8.5477E-06 | Hdac2,15182:up\|Msl2,77853:up\|Ctr9,22083:up\|Cbx6,494448:up\|Hist1h1e,50709:up\|Emsy,233545:up\|Rybp,56353:up\|Brd7,26992:down\|Kdm3a,104263:up\|Ubr5,70790:up\|Actb,11461:down\|Brd2,14312:down\|Brd3,67382:up\|Rbbp4,19646:up\|Kdm1a,99982:up\|Chd7,320790:down\|Huwe1,59026:up\|Wdr5,140858:up\|Tet3,194388:down\|Actl6a,56456:up\|Setd2,235626:up\|Elk4,13714:up\|Kat6b,54169:up\|Usp16,74112:up\|Arid1a,93760:up\|Atrx,22589:up\|Rnf20,109331:up\|Skp1a,21402:down\|Kdm6a,22289:up\|Gatad2b,229542:up\|Trip12,14897:up\|Baz1b,22385:up\|Nap1l1,53605:up\|Tet2,214133:up\|Brcc3,210766:up | http://amigo.geneontology.org/amigo/term/GO:0006325 |
